# Enhanced Therapeutic Efficacy of Lispro-Protamine Insulin Via Vanadate and Decavanadate Functionalization in a Type 1 Diabetes Murine Model

**DOI:** 10.1007/s12011-025-04966-7

**Published:** 2026-01-07

**Authors:** Ulises Peña-Rosas, Anahí Cruz-Cuateco, Diana Moroni-González, Lisset Noriega, Alfonso Díaz, Rubén Antonio Vázquez-Roque, Eduardo Brambila, Enrique González-Vergara, Samuel Treviño

**Affiliations:** 1 Faculty of Chemistry Science, Meritorious Autonomous University of Puebla, 22 South. FC91, University City, Puebla, C.P. 72560 Mexico; 2 Laboratory of Chemical-Clinical Investigations, Department of Clinical Chemistry, Faculty of Chemistry Science, Meritorious Autonomous University of Puebla, 14 South. FCQ1, University City, Puebla, C.P. 72560 Mexico; 3 Laboratory of Metabolomic and Chronic Degenerative Diseases, Physiology Institute, Meritorious Autonomous University of Puebla, Prol. de la 14 Sur 6301, Ciudad Universitaria, Puebla, C.P. 72560 Mexico; 4 Department of Applied Physics, Center for Research and Advanced Studies of the National Polytechnic Institute, Mérida, 97205 Mexico; 5 Laboratory of Neurochemistry and Behavior, Physiology Institute, Meritorious Autonomous University of Puebla, Prol. de la 14 Sur 6301, Ciudad Universitaria, Puebla, C.P. 72560 Mexico; 6 Laboratory of Neuroplasticity and Metabolism, Physiology Institute, Meritorious Autonomous University of Puebla, Prol. de la 14 Sur 6301, Ciudad Universitaria, Puebla, C.P. 72560 Mexico; 7 Laboratory of Applied Bioinorganic. ICUAP Chemistry Center, Meritorious Autonomous University of Puebla, 22 South. FC91, University City, Puebla, C.P. 72560 Mexico

**Keywords:** Lispro-protamine, Insulin, Diabetes, Vanadate, Decavanadate

## Abstract

Diabetes is a complex condition that requires continuous medical care and risk-reduction strategies. Therefore, technological improvements and the development of new insulins are necessary. This work aimed to develop insulin functionalized with vanadate (V1) or decavanadate (DV10) and probe its effectiveness on glucose and lipid homeostasis in a like-T1D murine model. Seven different molar combinations of HUMALOG ^®^ 75/25 insulin with V1 or DV10 were characterized using molecular docking, UV/visible, and FT-IR spectroscopy, confirming stable interactions between V1 or DV10 with the insulin. Then, a like-T1D model was generated in Wistar rats. The acute pharmacodynamics and pharmacokinetics of functionalized insulin were evaluated. Ins-V1 9:1 and 7:3 ratios demonstrated functional bioequivalence to commercial Lispro insulin, reducing glycemia by approximately 67.7% and 60%, within 60 min. The ins-DV10 3:7 and 5:5 ratios exhibited lower average glycemia than Lispro (*p* < 0.01), indicating greater cumulative efficacy. In the long term (30 days), the ins-V1 and ins-DV10 combination administered successfully reversed hyperglycemia and dyslipidemia. Treatments enhanced glycogen storage by 50%–80% and reduced tissue steatosis by 22%–62.5% in the liver, muscle, heart, and kidney. Ins-DV10 (3:7 and 1:9) were better than any ins-V1 combination. Remarkably, acute V1 treatment alone improved dyslipidemia and hyperglycemia, but in the long term, DV10 alone showed the greatest efficacy in both serum and tissues. In conclusion, functionalization with a higher proportion of DV10 significantly enhances the pharmacokinetics and pharmacodynamics of lispro-protamine insulin. Therefore, it could be considered a treatment strategy for managing glucose and lipid homeostasis in diabetes.

**Supplementary Information:**

The online version contains supplementary material available at 10.1007/s12011-025-04966-7.

## Introduction

Diabetes is a complex, chronic condition that requires continuous medical care and comprehensive risk-reduction strategies; it has reached pandemic proportions, with an overall prevalence of 10–20% in recent decades [1]. The American Diabetes Association’s professional committee defines it as a metabolic disorder of carbohydrate metabolism in which glucose is underutilized as an energy source. This causes hyperglycemia, induced by a lack of insulin or loss of its signaling in tissues [2]. Insulin is an anabolic peptide hormone secreted by β-cells of the pancreas. After binding its receptor in the cell membrane of tissues such as the liver, heart, skeletal muscle, and kidney, it exerts its effects. Insulin signaling controls glucose uptake, glycogenesis, glycogenolysis, gluconeogenesis, protein synthesis, lipolysis, free fatty acids (FFA) uptake, lipogenesis, and, at least in part, beta-oxidation by the metabolic arm. Besides, by the mitogenic arm, insulin controls cellular growth, differentiation, apoptosis, and proliferation [3–5].

Type 1 diabetes (T1D), type 2 diabetes (T2D) patients, and some specific cases of diabetes eventually require insulin replacement therapy. In TD1 subjects, autoantibodies against Langerhans islets destroy them, leading to insulin deficiency; these subjects require insulin therapy. Meanwhile, T2D subjects exhaust Langerhans islets due to continuous hyperinsulinemia and insulin resistance, thereby requiring long-term exogenous insulin administration [2]. Since its discovery in 1921, insulin has been the standard treatment for complicated hyperglycemia. For many years, insulin from cattle and pigs was used to treat diabetes, but it caused allergic reactions in many patients. In 1978, using *E. coli* bacteria, the first genetically engineered, synthetic “human” insulin was produced. Humulin was sold as the first commercially available biosynthetic human insulin [6, 7]. Currently, exogenous insulin comes in many forms, ranging from regular human insulin, identical to what the body produces, to ultra-rapid and ultra-long-acting insulins [6, 8]. Pharmaceutical development has focused on long-acting products to avoid the necessity of multiple daily injections, thereby enhancing patient convenience and mitigating the instability of insulin solutions, especially in the absence of zinc-mediated self-assembly [9]. However, the pharmaceutical industry is seeking novel insulin formulations that are more efficient and cost-effective.

Insulin mixes (e.g., short-acting or rapid-acting mealtime insulin with intermediate-acting or long-acting insulin to maintain glycemia) limit daily injections, providing greater coverage throughout the day in basal and postprandial conditions and avoiding hyperglycemic peaks. Humalog^®^ Mix 75/25 is a lispro and lispro-protamine insulin mix containing intermediate- and fast-acting insulin, a well-established treatment for various types of diabetes [8, 10]. Furthermore, commercially available concentrated insulins deliver more insulin with less volume and improve treatment adherence [9, 11]. These involve the acylation of insulin’s unique side-chain amino group on lysine at position 29 (or replacement by glutamic acid) of the B chain in a zinc insulin analog hexamer, stabilizing insulin depot formation and mediating binding to albumin, thereby delaying clearance from the bloodstream [12, 13]. This conformational change enables a linear array of hexamers (“molecular serendipity”) rather than the classical pairwise bridging observed in the formulated dimer of hexamers [9]. Considering Humalog Mix 75/25’s advantages and serendipity stability, we designed a modified, insulin-functionalized, and stabilized form by metavanadate or decavanadates, which theoretically possesses fast-acting and ultra-lente insulin action without bioequivalency complications.

The biological role of vanadium compounds as insulin-action enhancer agents has been established [3, 4, 14–18]. Vanadium inorganic and organic salts have been extensively explored for the treatment of metabolic diseases, such as diabetes. Moreover, pharmacological polyoxidovanadates (POVs) actions, such as antidiabetic, antibacterial, antiprotozoal, antiviral, and anticancer drugs, have been tested [19, 20]. Particularly, decavanadate (DV10 or V10), a POV so widely studied, is primarily associated with its ability to mimic phosphate and inhibit or activate specific enzymes involved in phosphorylation and energy metabolism, such as protein tyrosine phosphatase 1B, but it also activates AMP-activated protein kinase [21, 22]. These pathways improve energy sense and insulin signaling, thus positively affecting glucose and lipid homeostasis. Additionally, they influence glycogen synthesis and adequate triglyceride storage in multiple tissues [17, 18]. Decavanadate is thermodynamically unstable and pH-dependent; however, at physiological pH, it decomposes slowly and, as a polyanion, can protonate with different counterions, which can also significantly influence stability [3, 4, 22]. Therefore, the general aim was to develop insulin functionalized with vanadate and decavanadate, use insulin as a counterion, in different equimolar combinations, and, as a particular aim, probe its effectiveness on glucose and lipid homeostasis in a like-T1D murine model.

## Materials and Methods

### Insulin Vanadium-Functionalized

All chemicals used were of reagent grade and were purchased from Sigma-Aldrich (Toluca, Mexico). The ammonium decavanadate solution was prepared by heating a 0.468 g (4 mmol) mixture of ammonium metavanadate in 18 mL of distilled water in an Erlenmeyer flask with magnetic stirring to 70 °C until dissolved. Then, four drops of concentrated hydrochloric acid (37%) at room temperature were added to allow decavanadate anion formation at pH 6 [16, 17, 23].

Separately, in free-metal Eppendorf tubes, 0.05 g of Chelex was hydrated with 1 mL of Tris-HCl (5 mM) at pH 8.4 and left in constant rotation for 8 h. Then, it was centrifuged at 300 x g for 20 min to remove the liquid phase. To the hydrated Chelex beads, HUMALOG ^®^ 75/25 insulin (1 mL) was added, and the mixture was stirred for 24 h to produce metal-free Insulin (Apo-insulin). Then, the apo-insulin was separated in free-metal Eppendorf tubes, and a molar concentration of V1 or DV10 was added. Mixes were left in constant rotation for 24 h, at 8 °C and pH 5.7, to form insulin-functionalized. Insulin was functionalized with a solution of decavanadate or ammonium metavanadate (0.17 mM) in molar conditions as follow: **(a)** HUMALOG^®^ 75/25; **(b)** 9 insulin moles + 1 decavanadate/vanadate mol (500 µL of insulin + 40 µL of decavanadate/vanadate; **c)** 7 insulin moles + 3 decavanadate/vanadate moles (500 µL of insulin + 156 µL of decavanadate/vanadate); **d)** 5 insulin moles + 5 decavanadate/vanadate moles (500 µL of insulin + 365 µL of decavanadate/vanadate); **e)** 3 insulin moles + 7 decavanadate/vanadate moles (500 µL of insulin + 849 µL of decavanadate/vanadate); **f)** 1 insulin mol + 9 decavanadate/vanadate moles (100 µL of insulin + 3278 µL of decavanadate/vanadate); **g)** Decavanadate/vanadate solution. It is important to note that metavanadate ions are present only in the solid state. At pH close to 7 and slightly higher, the species most likely to interact with insulin are V1 and V4, with V4 showing stronger binding to proteins.

### Insulin Vanadium Characterization

UV/visible spectroscopic characterization was recorded on a Thermo Scientific Multiskan SkyHigh spectrophotometer (Thermo Fisher Scientific, Marietta, OH). 1 mM of insulin, DV10, V1, insulin-V1 (Ins-V1), and insulin-DV10 (Ins-DV10) solutions, in the injection dilution medium containing 0.9% NaCl, pH 5.7, at 25 °C, were analyzed. For each experiment, 1.5 mL of each solution was placed in a quartz cuvette (1 cm path length) and continuously excited with UV light. A fresh sample was used for each excitation session. Samples were magnetically stirred at 100 x g to ensure homogeneous excitation. Slits (bandpass) were always set to 5 nm. Lamp power was 142 µW, and the same lamp was used throughout the experiments. Absorbance spectra were acquired between 200 and 800 nm. Band assignments to insulin, DV10, V1, insulin-V1 (Ins-V1), and insulin-DV10 (Ins-DV10) solutions were according to the literature [24, 25].

Under the same conditions as for UV/visible spectroscopy, samples were analyzed by infrared spectroscopy, with spectra obtained in solution from 400 to 4000 cm^− 1^ using an IR Digilab Model Scimitar FT-IR spectrophotometer (Varian Inc., USA). For commercial insulin, all excipients were purchased at analytical grade (Sigma-Aldrich, St. Louis, USA) and prepared with known concentrations for insulin-free infrared background measurements. Absorbance spectra of insulin and reference excipients were recorded with a resolution of 4 cm^− 1^, and Fourier transformation was applied. The total measurement time was approximately 2 min, resulting in 400 sample interferogram scans at ambient temperature. Water-absorbance-compensated spectra were calculated using buffer solution or distilled water as the spectral background. For the analysis of the insulin characteristic amide I band between approximately 1700 and 1600 cm^− 1^, second-derivative spectra were calculated after min–max normalization of the absorbance spectra to localize the positions of the secondary structure single bands. For the determination of structural changes within the insulin molecule in an aqueous sample, it is well-suited; thus, the maximum of the amide I band is at 2 absorbance units in the wavenumber range of 1680–1450 cm^− 1^. Bands I and II were monitored by the decrease in the intensity of the water absorption band around 1650 cm^− 1^. The amides II and III band positions were determined at 1525 cm^− 1^ and 1356 cm^− 1^. These analysis conditions were also applied to insulin-V1 and insulin-DV10 formulations. The infrared spectroscopy analysis conditions for ammonium metavanadate and decavanadate were realized as previously informed [16, 26, 27]. All manipulations were conducted at room temperature, with no special solvent and reagent purification.

### Docking Methodology

Molecular docking analysis was performed using the semi-flexible method, in which the protein fragment (PDB ID 5udp) was treated as a rigid entity, while the ligand DV10 was allowed to be flexible. The macromolecule and ligand were prepared using Autodock Tools 1.5.6, which includes polar hydrogens and empirical atomic charges (Gasteiger–Marsili method). The grid box dimensions were 126 Å × 126 Å × 126 Å to enclose the entire protein. The grid spacing for all docking calculations was set to the default 0.375 Å value, using the Lamarckian genetic algorithm (LGA) search method, with 200 GA runs and a maximum of 1 top individual automatically surviving. The parameters for the vanadium atom were the sum of the VDW radii of two similar atoms (3.14 Å), plus the VDW well depth (0.016 kcal/mol), plus the atomic solvation volume (12.0 Å3), plus the atomic solvation parameter (− 0.00110). The H-bond radius of the heteroatom in contact with hydrogen (0.0 Å), the well depth of the H-bond (0.0 kcal/mol), and different integers indicate the H-bonding atom and indexes for the generation of the autogrid map (0, − 1, −1, 1, respectively).

### Biological Studies

140 male Wistar rats (300–320 g) obtained from the vivarium Claude Bernard of the Universidad Autónoma de Puebla were used and maintained under a climate-controlled environment, 12 h light-dark cycles, a temperature of 19–26 °C, and availability of food and water *ad libitum*. Animals were conditioned with a regular calorie diet (5001, LabDiet; St. Louis, MO, USA). The animals were randomly assigned using a random number generator in Excel; the procedure was performed by the veterinary staff in charge of the rats to ensure blinding. Three groups were formed as follows: (1) No-treatment type 1 diabetes group (NT-T1D; *n* = 10); (2) HUMALOG^®^ 75/25 insulin-treated group (Lispro insulin; *n* = 10); and (3) decavanadate/vanadate group (*n* = 120). Then, alloxan (150 mg/kg) was administered intraperitoneally. Serum glucose and insulin concentration were monitored starting the third day after alloxan administration. When hyperglycemia (above 300 mg/dL) and hypoinsulinemia (close to 0 µUI/mL) were observed, the like-type 1 diabetes (T1D) model was validated [14]. The animals in group 3 were again randomly assigned to 12 working groups (*n* = 10 each), of which 6 received treatments with ammonium metavanadate or ins-V1 combinations, and 6 received treatments with DV10 or ins-DV10 combinations under different molar conditions, as indicated in Sect. 2.1. The NT-T1D group did not receive treatment, and the Lispro insulin group was initially administered subcutaneously at a dose of 5 U/day. All procedures described in this study were approved by the ethics committee CICUAL-BUAP for animal handling. The Guide for the Care and Use of Laboratory Animals of the Mexican Council for Animal Care NOM-062-ZOO-1999 was followed for each procedure described in this study. All applicable international (ARRIVE 2.0 and NHI guidelines), national, and institutional guidelines for the care and use of animals were followed to minimize possible discomfort.

###  Evaluation of the Insulin Activity

The T1D rats were administered lispro insulin (HUMALOG 75/25 insulin, Eli Lilly and Company, USA) for three days, or a molar composition of metavanadate- or decavanadate-functionalized insulin at a dose of 5 U/day subcutaneously; the dose was later adjusted according to glycemic levels. The T1D rats received insulin doses at a fixed time (9:00 AM) every day until euthanasia. On the first day, lispro-protamine insulin activity was evaluated by glucose monitoring with a glucometer (ACCU-CHEK Active, Roche Diagnostics, Germany) and by insulin measurement using a commercial kit and a CL900i chemiluminescence autoanalyzer (DESEGO, Michoacán, Mex). Blood samples were obtained from the tail vein at −15, 0, 15, 30, and 60 min after insulin administration. On the second day, lispro insulin intermediate activity was evaluated by measuring serum glucose and insulin concentrations from tail-vein samples obtained every 2 h for 12 h after insulin administration. On the third day, 1 h after treatment administration, five rats from each group were anesthetized with ketamine and xylazine (0.2 ml/100 g, intraperitoneally). 1 mL of blood was collected by intracardiac puncture with a BD Vacutainer Venous Blood Collection system in tubes with and without K_3_EDTA. Samples were centrifuged at 400 x g for 10 min; the serum and plasma were separated and frozen at − 70 °C until analysis. Rats were immediately sacrificed and perfused with cold isotonic saline. Biopsies from tissues (liver, heart, kidney, and muscle) were taken and stored at − 70 °C for subsequent determination of glycogen, triglyceride, and vanadium quantification.

The remaining T1D rats continued their treatments for 30 days. Fasting glucose and insulin were measured daily. An oral glucose tolerance test (OGTT) was performed at the end of the experiment. The anhydrous glucose load was administered orally via gavage (1.75 g/kg body weight). Then, the animals were anesthetized (with ketamine + xylazine at 0.2 mL/100 g, intraperitoneally). Blood samples were collected by intracardiac puncture (200 µL) in tubes without anticoagulant for serum separation. Blood was collected at 0, 30, 60, and 90 min, and the samples were immediately centrifuged at 400 x g for 10 min. 1 mL of blood was collected at 90 min in tubes with and without K_3_EDTA. The serum and plasma were separated and frozen at − 70 °C until analysis. For fasting blood samples (time 0 min), food and water were withheld for 4–5 h before collection. Biopsies from the liver, muscle, heart, and kidney were obtained. The tissues were perfused with cold isotonic saline and stored at − 70 °C.

###  Biochemical Assays

In each sacrifice, biochemical parameters were analyzed. The concentrations in serum of glucose, fructosamine, triglycerides (TG), total cholesterol (TC), Low-Density Lipoprotein-Cholesterol (LDL-C), and High-Density Lipoprotein-Cholesterol (HDL-C) were measured using an A15 autoanalyzer (BioSystems, Guadalajara, Mexico). Very low-density lipoprotein (VLDL) concentration was obtained using the Martin-Hopkins estimation [5]. Serum biomarker assays for hepatic and renal damage were performed in animals treated for 1 month and measured using an A15 autoanalyzer (BioSystems, Guadalajara, Mexico; renal profile: urea, creatinine, sodium, potassium; hepatic profile: aspartate aminotransferase [ASAT], alanine aminotransferase [ALAT], alkaline phosphatase [ALP], and total bilirubin), see supplementary Table S2. Insulin measurement using a commercial kit and CL900i chemiluminescence autoanalyzer (DESEGO, Michoacán, Mex). The free fatty acids (FFAs) were extracted with chloroform, assessed with the reagent copper-cuprizone, and detected with an ammonia source; the concentration was determined with a PerkinElmer Lambda EZ150 spectrophotometer at 620 nm [5]. Additionally, from biopsies, 100 mg of tissue was homogenized in 800 µL of ISS; the homogenate was used for TG determination following the manufacturer’s instructions. To determine glycogen, we used the technique described by Bennett in 2007 [28, 29].

### Quantification of Vanadium in Plasma and Tissues

The concentration of vanadium was determined using an inductively coupled plasma optical emission Spectrometer (Mod. 730-ES, Varian Inc., USA). Five hundred milligrams of muscle, liver, heart, renal cortex, and renal medulla from each experimental group were placed in a solution of nitric acid (50%) and perchloric acid (15.6 M) in a 1:1 ratio. The mixture was incubated at 37 °C for three days in an ACCU BLOCJTM bath (Labnet International, USA) until complete digestion. Then, the sample was diluted with deionized water to a final volume of 5 mL. Deionized water (resistivity 18.2 MΩ cm) was dispensed from a Barnstead Easypure II water system (Thermo Fisher Scientific, Marietta, OH). Plasma samples were prepared: 0.25 mL of plasma was aliquoted into an acid-washed glass centrifuge tube and mixed with 2.25 mL of the eluent. Samples were then gently mixed by vortex and centrifuged at 3500 x g for 7 min. Supernatants were transferred to 10 kDa cutoff filter tubes and centrifuged again at 3500 x g for 20 min, and the resulting filtrate was analyzed. The inductively coupled plasma optical emission spectrometer was calibrated using two tuning solutions: a mixed-stock standard and an internal standard solution. Six-point calibration standards were generated over suitable ranges. All laboratory materials, such as the micropipette tips, tubes, and vials, were cleaned by an overnight soak in 20% v/v HNO_3_ and rinsed with deionized water.

###  Statistical Analysis

Data are expressed as mean ± standard error of the mean (SEM). Normality of the distributions was assessed using the Shapiro–Wilk test. For comparisons among groups, one-way analysis of variance (ANOVA) was performed, followed by the Dunnett post hoc test. For multi-time-point data (glucose and insulin curves), a Two-Way Repeated Measures ANOVA was conducted to evaluate treatment effects, followed by Tukey’s post hoc test. All statistical analyses were carried out using GraphPad Prism version 8 (GraphPad Software Inc., USA). Differences were considered statistically significant at *p* ≤ 0.05. Statistical comparisons were performed against the Lispro insulin group, which served as the treated control.

## Results

### Insulin-Functionalized Characterization

**Fig. 1:**
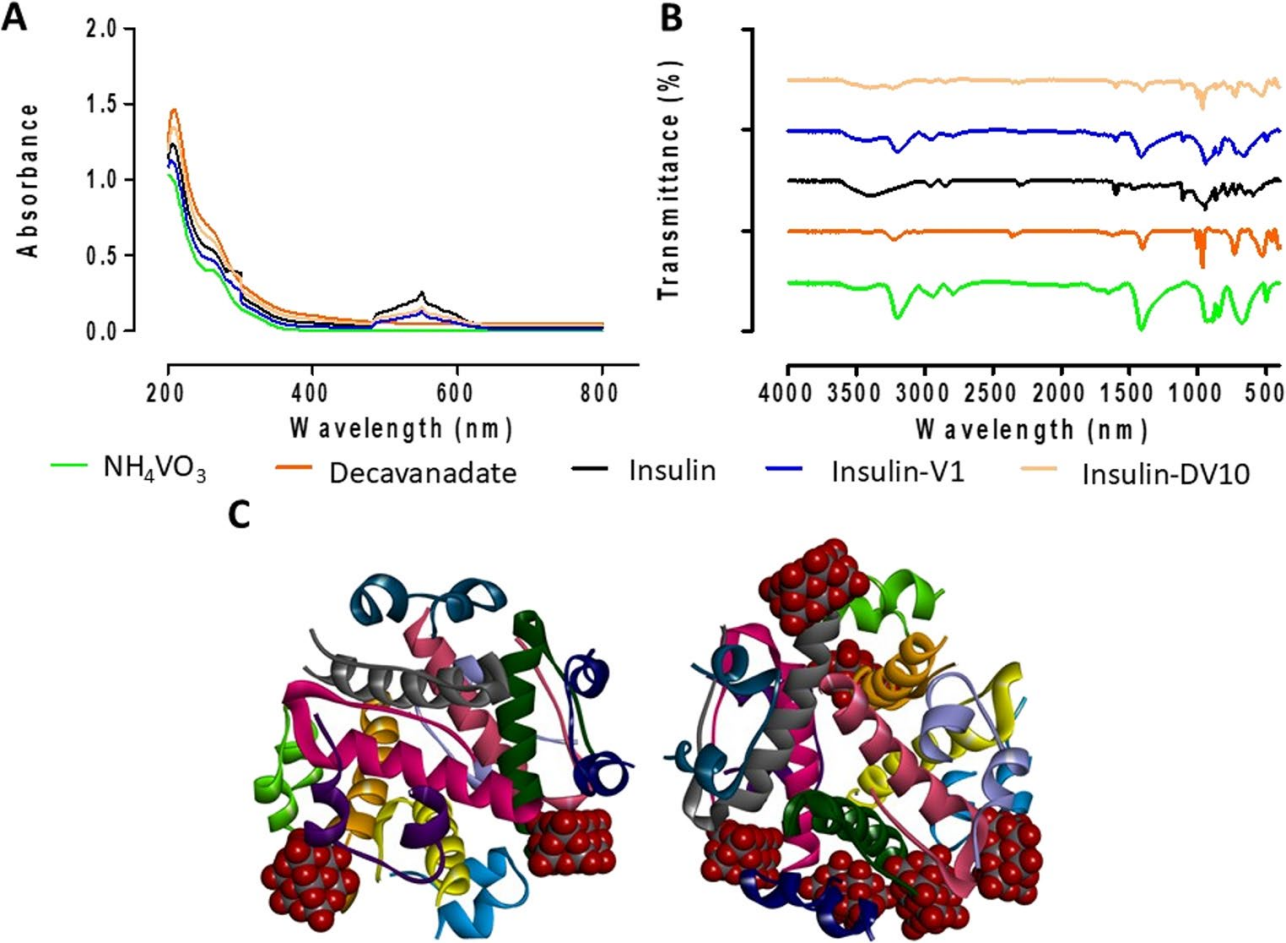
Insulin-functionalized characterization. **(A)** UV/visible analysis. **(B)** FT-IR analysis. **(C)** Molecular docking. Decavanadate and Lispro insulin hexamer interactions. Chain A: Purpure, Chain B: pink, Chain C: Green, Chain D: orange, Chain E: blue, Chain F: gray, Chain G: dark blue, Chain H: dark green, Chain I: cyan, Chain J: gold, Chain K: pale blue, Chain L: pale red

The ammonium metavanadate, decavanadate, insulin, and functionalized insulin with metavanadate and decavanadate (5 insulin moles + 5 decavanadate/vanadate moles) showed one shoulder with very low intensity at about 245 nm, attributed to the charge transfer vanadium-oxygen. HUMALOG^®^ insulin and functionalized insulin with V1 and DV10 show an increase in absorbance intensity at 240–285 nm (where Trp, Phe, Tyr, and His absorb), and a second peak at about 545 nm, assigned to insulin aggregates or polymers (hexamers). Additionally, the decavanadate exhibited a characteristic shoulder at about 400 nm, which is used to evaluate its dissociation kinetics (Fig. 1A). Figure 1B shows the IR spectrum of ammonium metavanadate at the bottom (green). The NH_4_VO_3_ showed a single N—H stretching mode at 3226 cm^− 1^ in addition to an N—H bending mode at 1433 cm^− 1^. The bands at 952 and 881 cm^− 1^ were assigned to V = O stretching modes, while the band at 715 cm^− 1^ was assigned to the O—V—O terminal stretching mode. The band at 507 cm^− 1^ can be assigned to the V—O bridging stretching modes. The decavanadate IR spectrum (red line) showed a single N—H stretching mode at 3280 cm^− 1^, an N—H bending mode at 1334 cm^− 1^, and an O—H bending mode at 1660 cm^− 1^. The bands at 1010 and 1008 cm^− 1^ were assigned to V = O stretching modes, and the band at 746 cm-1 was assigned to the V–O–V bridging antisymmetric vibrations. The bands at 565–501 cm^− 1^ were assigned to the V–O–V in-plane bending modes.

The amide I, II, and III bands of insulin are readily detectable and can be used to analyze its secondary protein structure. As a first step, the absorbance spectra were preprocessed by minimum-maximum normalization between 1780 and 1580 cm^− 1^, limiting the maximum of the amide I band to 2 absorbance units. For the insulin samples, a shift was observed in the amide A band, assigned to the N—H stretching vibration, to around 3440 cm^− 1^. The amide I band was observed at 1745 cm^− 1^, indicative of alpha-helical secondary structure. The spectrum of the insulin fibrils showed a significant shift of the most intense band around 1622 cm^− 1^ and a distinct shoulder around 1680 cm^− 1^. These bands can be assigned to the parallel beta-sheet and turn substructures of insulin fibrils. The amides II and III band positions were determined at 1525 cm^− 1^ and 1356 cm^− 1^, respectively. The leading excipients of lispro insulin are phenol and glycerol, with band positions at 1114 cm^− 1^ and 929 cm^− 1^, respectively. In the functionalized insulins were observed an N—H bending mode at 1440 cm^− 1^ (ins-V1, blue line) and 1440 cm^− 1^ (ins-DV10, orange line), the bands at 958 (ins-V1) and 970 cm^− 1^ (ins-DV10) were assigned to V = O stretching modes, while the bands at 715 (ins-V1) and 742 cm^− 1^ (ins-DV10) were given to the V–O–V bridging antisymmetric vibrations, and the band at 550 cm^− 1^ of ins-V1 can be assigned to the V–O–V in plane bending modes, while ins-DV10 showed it around 588 cm^− 1^ (Fig. 1B).

In addition, a docking analysis was performed using 200 Genetic Algorithm-generated poses to explore all possible interaction sites between decavanadate and lispro insulin. Table 1 reports the top 6 poses of DV10 interacting with different chains. The binding energies ranged from − 10.27 to −9.68 kcal/mol. The interactions involve van der Waals and hydrogen bonds with Gln4, Glu21, and Leu13 from different protein chains. Two DV10 clusters interact with both the A and B chains of insulin. A hexamer of insulin can bind six DV10 clusters, forming a nanocompound with a specific distribution. In Fig. 1C (left), two DV10 bind to the protein similarly. In Fig. 1C (right), six DV10 are observed to interact with the protein; in silico studies also revealed that the most probable speciation of vanadate is tetravanadate (cyclotetravanadate; Supplementary Table 1 and Fig. 1). NMR studies will be necessary in the future to elucidate insulin-vanadium speciation.

**Table 1:** Docking results. Binding energies for best molecular poses of decavanadate in complex with lispro insulin

Compound	Target	Binding Energies (Kcal/mol)	Interactions
Decavanadate + Lispro Insulin	Chain E and I	**−10.27**	Hydrogen bond (3), Tyr14, Leu13, Val18, Arg22, Glu17, Glu21, Gly20,
	Chain B, G, H	**−10.21**	Hydrogen bond (2), Phe1, Gln4, Asn3, Cys7, Thr8, Asn3, Val2
	Chain I and L	**−9.94**	Hydrogen bond (2), Glu21, Gly20, Cys19, Leu17, Ser12, Leu13, Tyr14, Arg22, Val18
	Chain H, G, L	**−9.87**	Hydrogen bond (1), Glu21, Thr27, Phe1, Gln4, Val3, Gly8, Tyr26, His5, Tyr16, Ile10
	Chain F	**−9.75**	Hydrogen bond (4), Asn3, Val2, Phe1, Gln4
	Chain B and F	**−9.68**	Hydrogen bond (2), Ile10, His5, Gly20, Lys28, Glu21, Pro29, Phe1, Gln4

The docking analysis considered 200 poses of the Genetic Algorithm. Decavanadate and Lispro insulin interactions involve van der Waals interactions and hydrogen bonds

### Biological Tests

**Fig. 2:**
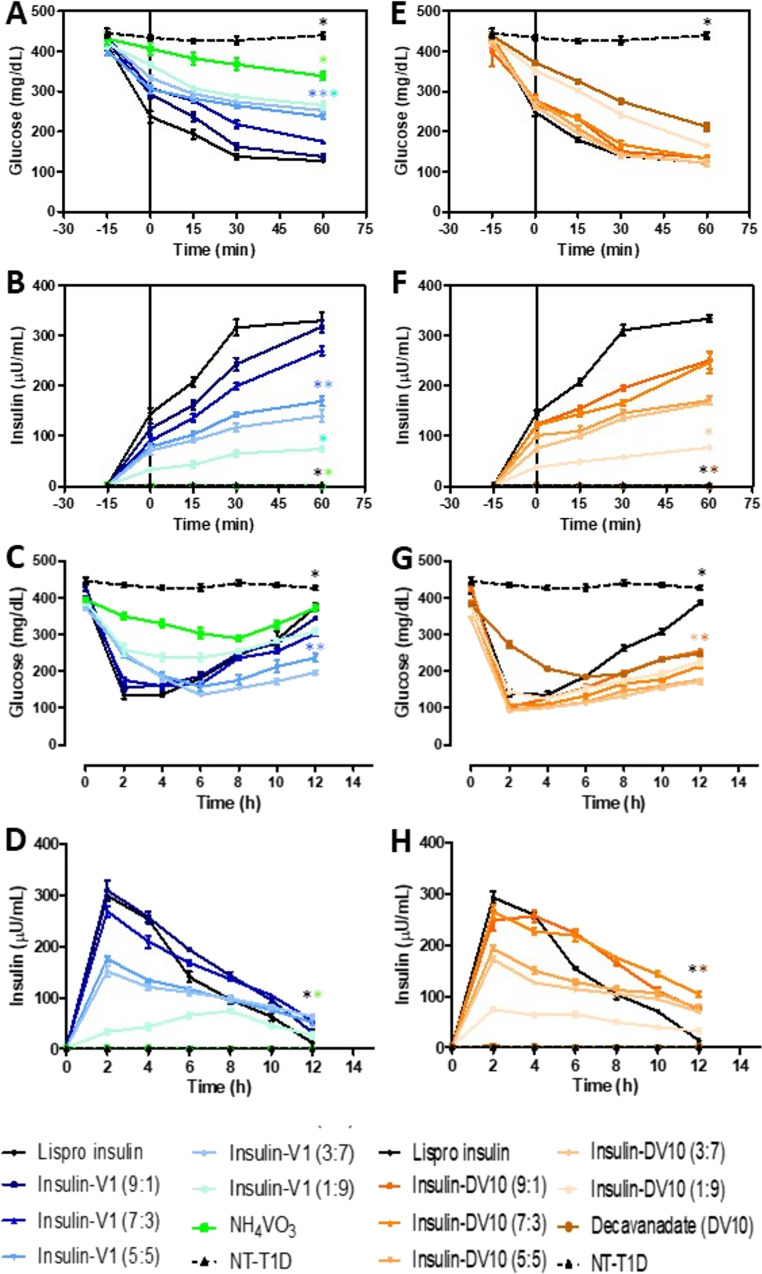
Acute pharmacodynamics and pharmacokinetics of insulin-functionalized. **(A)** Pharmacodynamics of insulin-vanadate molar combinations, lispro activity evaluation. **(B)** Pharmacokinetics of insulin-vanadate molar combinations, lispro activity evaluation. **(C)** Pharmacodynamics of insulin-vanadate molar combinations, lispro-protamine activity evaluation. **(D)** Pharmacokinetics of insulin-vanadate molar combinations, lispro-protamine activity evaluation. **(E)** Pharmacodynamics of insulin-decavanadate molar combinations, lispro activity evaluation. **(F)** Pharmacokinetics of insulin-decavanadate molar combinations, lispro activity evaluation. **(G)** Pharmacodynamics of insulin-decavanadate molar combinations, lispro-protamine activity evaluation. **(H)** Pharmacokinetics of insulin-decavanadate molar combinations, lispro-protamine activity evaluation. The results shown are the average of 5 separate experimental animals ± SEM. (*) Indicates a significant difference from the Lispro insulin group. *P* ≤ 0.05 by two-way Repeated Measures ANOVA was conducted to evaluate treatment effects, followed by Tukey’s post hoc test

After inducing hyperglycemia (upper 400 mg/dL) and hypoinsulinemia (below 2 µU/mL), the TD1 model was validated (dotted line, Fig. 2A-B). The lispro (fast-acting) and lispro-protamine (intermediate-acting) activity was verified. Five units of HUMALOG^®^ 75/25, NH_4_VO_3_, and DV10 functionalized insulin were applied to T1D rats. Lispro insulin (control group) reduced glycemia by 43.2% (from 416.7 to 236.7 mg/dL), 53.7% (193 mg/dL after 15 min), 67.2% (136.7 mg/dL after 30 min) and 69.7% (126.3 mg/dL after 60 min) (Fig. 2A). Equimolar combinations of ins-V1 showed a hypoglycemic effect (F_(7,28)_ = 18.56; *p* < 0.0001) after 15 and 60 min by 5% and 21.6% (NH_4_VO_3_), 12.6% and 36.7% (ins-V1; 1:9), 21.7% and 40.8% (ins-V1; 3:7), 24.1% and 41.1% (ins-V1; 5:5), 29.5% and 60% (ins-V1; 7:3), 31.1% and 67.7% (ins-V1; 9:1). The formulations with 9:1 and 7:3 ratios showed no statistically significant differences compared with Lispro insulin (*p* = 0.9984 and 0.8421, respectively). This suggests that these Ins-V1 combinations replicate the hypoglycemic potency of commercial insulin (functional bioequivalence). Plasma insulin levels increased progressively with increasing equimolar combinations (F_(7,28)_ = 12.18; *p* < 0.0001), indicating that high vanadate concentration altered the velocity and insulin release peak. Ins-V1 combinations 9:1 and 7:3 showed a release kinetics similar to Lispro. Meanwhile, the control group (Lispro insulin) observed a mean of 145 µU/mL after 15 min of administration (at 0 min), 208.3 µU/mL (at 15 min), 316.3 µU/mL (at 30 min), and 330 µU/mL (at 60 min). The ins-V1 (1:9), NH_4_VO_3_, and NT-T1D groups differed significantly from the control group (Fig. 2B).

Equimolar combinations of ins-DV10 also observed a hypoglycemic effect according to insulin concentration (F_(7,28)_ = 22.14; *p* < 0.001). Hypoglycemic effect was observed after 15 and 60 min by 15.3% and 51.7% (DV10), 18.6% and 62% (ins-DV10; 1:9), 38.3% and 71.4% (ins-DV10; 3:7), 33.5% and 71.3% (ins-DV10; 5:5), 39.5% and 69.7% (ins-DV10; 7:3), 30.3% and 66.4% (ins-DV10; 9:1). However, glycemia was more homogeneous across the treated groups with the ins-DV10 combinations 5:5, 7:3, and 9:1, and there were no differences in the Lispro insulin group, which indicates that they possess a similar efficiency to commercial insulin (Fig. 2E). However, insulin concentrations were lower with ins-DV10 combinations than with commercial treatment, suggesting slower pharmacokinetics that depend on hormone content. The plasma insulin levels in the ins-DV10 9:1 and 7:3 formulations did not differ from those of commercial insulin, indicating similar bioavailability (*p* > 0.05). Therefore, the ins-DV10 combinations 5:5, 3:7, and 1:9, as well as the DV10 and NT-T1D groups, were statistically different from the control group (Fig. 2F).

The lispro-protamine activity was monitored for 12 h (Fig. 2C). The lispro-protamine reduced glycemia by 65.5% (from 394 to 136 mg/dL after 2 h), 65.4% (136.3 mg/dL after 4 h), 54% (181.3 mg/dL after 6 h), 38.2% (243.7 mg/dL after 8 h), 27.4% (286 mg/dL after 10 h), and 5.2% (373.7 mg/dL after 12 h); this group showed a maximal hypoglycemic effect from 2 to 4 h (136 mg/dL) and a slow return to hyperglycemia at 12 h (371 mg/dL). The hypoglycemic effect of NH_4_VO_3_ was 11.6%, 16.5%, 23.3%, 26.7%, 17.1%, and 5.8% at 2, 4, 6, 8, 10, and 12 h, respectively. For ins-V1 (1:9), the hypoglycemic effect was 33.6%, 38.8%, 39.2%, 34.9%, 27.5%, and 20.7%, respectively. For ins-V1 (3:7), the effect was 33.2%, 51.1%, 63.6%, 58.7%, 53.7%, and 47.2%, respectively. For ins-V1 (5:5), the effect was 37.6%, 51.7%, 59.2%, 54.8%, 45.3%, and 39.1%, respectively. For ins-V1 (7:3), the effect was 55.8%, 59.8%, 58.5%, 40.6%, 35.9%, and 23.9%, respectively. Meanwhile, for ins-V1 (9:1), the observed effect was 64.2%, 62.1%, 56.8%, 42.1%, 35.8%, and 20.2%, respectively. Two-way Repeated-Measures ANOVA indicates a significant interaction between groups and treatments (F_(7,42)_ = 22.93; *p* < 0.001), indicating that glycemia varies over time by treatment. The ins-V1 9:1, 7:3, 5:5 combinations were not different from Lispro-protamine treatment, suggesting similar hypoglycemic activity (*p* > 0.05). Equimolar combinations 5:5 and 3:7 had maximal effects at 6 h (164 mg/dL and 135 mg/dL, respectively), maintaining hypoglycemic activity up to 12 h (236 mg/dL and 196 mg/dL, respectively). The NH_4_VO_3_ treatment showed the most significant effect at 8 h (289 mg/dL), which was significantly higher than those of the commercial treatment and ins-V1 combinations (Fig. 2C). The insulin pharmacokinetic in the Lispro-protamine group was at 2 h (298 µU/mL), 4 h (298 µU/mL), 6 h (140 µU/mL), 8 h (95 µU/mL), 10 h (63 µU/mL), and 12 h (11.7 µU/mL). Equimolar combinations of ins-V1 compounds exhibited plasma insulin concentrations that varied over time and treatments (F_(7,42)_ = 75.37; *p* < 0.001). Particularly, ins-V1 (9:1), (7:3), (5:5), and (3:7) combinations have a similar pharmacokinetic profile to Lispro-protamine (*p* > 0.05). The insulin concentrations in the V1 and NT-T1D groups were below 2 µU/mL; therefore, they were statistically different from those in the Lispro-protamine group (Fig. 2D).

The hypoglycemic activity of DV10 and Lispro-protamine ins-DV10 combinations was also analyzed (Fig. 2G). DV10 improved glycemia by 28.6%, 45.9%, 51.7%, 49.5%, 39.1%, and 35.3% at 2, 4, 6, 8, 10, and 12 h, respectively. Ins-DV10 (1:9) improved by 61.9%, 66.8%, 59.7%, 53.5%, 48.5%, and 39.7%, respectively. Ins-DV10 (3:7) improved by 73.2%, 70.4%, 66.7%, 61.2%, 54.3%, and 49.7%, respectively. Ins-DV10 (5:5) improved by 74.2%, 72.7%, 69.3%, 61.2%, 57.5%, and 53.1%, respectively. Ins-DV10 (7:3) improved by 71.9%, 71.4%, 65.5%, 56.9%, 54%, and 44.3%, respectively. Meanwhile, ins-DV10 (9:1) improved by 73.6%, 70.8%, 63.9%, 54.5%, 45.8%, and 40.7%, respectively. The statistical analysis showed that the hypoglycemic response to DV-10 and ins-DV10 combinations varies notably over time under experimental conditions (F_(7,42)_ = 44.45; *p* < 0.001). The statistical analysis showed that the hypoglycemic response to DV-10 and ins-DV10 combinations varies notably over time under experimental conditions. Interestingly, although Lispro-protamine treatment had a better hypoglycemic effect during the first 6 h, DV10 treatment maintained a long-lasting effect from 6 h (184 mg/dL) to 12 h (247 mg/dL), and the two treatments did not differ statistically (*p* = 0.998). Ins-DV10 combinations 1:9, 7:3, and 9:1 did not show a statistical difference from commercial treatment (*p* > 0.05), indicating a hypoglycemic comparable potential. The ratios 3:7 and 5:5 of ins-DV10 showed significantly lower average glucose levels than Insulin Lispro (*p* = 0.0026 and *p* = 0.0095, respectively), suggesting greater cumulative efficacy or longer duration of action at these ratios in this experiment (Fig. 2G). The insulin functionalized with DV10 stabilized the hormone, prolonging its presence in the bloodstream compared with lispro-protamine treatment (F_(7,42)_ = 66.40; *p* < 0.001). The ratios 3:7 to 9:1 did not show statistically significant differences compared with Lispro insulin (*p* > 0.05), suggesting that these formulations provide insulin bioavailability similar to that of the commercial insulin. The ins-DV10 1:9 ratio was significantly lower than the 5:5 to 9:1 ratios (*p* < 0.05), confirming that a higher proportion of DV10 in the mixture reduces the amount of circulating insulin detected (Fig. 2H).

A serum panel of carbohydrates and lipids was analyzed in the fasting state on the third day to evaluate the residual effects of the treatments (12 h after administration). Lispro-protamine treatment maintains reductions in glucose (69.7%), fructosamine (24.4%), total cholesterol (23.5%), LDL-C (50.6%), HDL-C (11.8%), and FFA (47.1%), but increases TG (44.2%) and VLDL-C (40.7%). The V1 and ins-V1 combination showed less control of glucose and lipids, particularly at equimolar insulin levels. The NH_4_VO_3_ treatment improved glucose and LDL-C levels by 18.8% and 31.5% respectively, compared with the NT-T1D group, but glucose, LDL-C, and fructosamine concentrations were significatively greater compared to lispro-protamine treatment. Equimolar combinations of ins-V1 1:9 to 9:1 improved glycemia by 36.3% to 67.1%, although only ins-V1 at lispro-protamine level; total cholesterol by 18% to 24.6%, all similar to lispro-protamine treatment; LDL-C by 31.5% to 43.6%, but only 7:3 and 9:1 combinations with comparable efficiency to lispro-protamine level; FFA by 41.3% to 49.6%, all without difference from lispro-protamine treatment; fructosamine diminished with 7:3 and 9:1 combinations similarly to lispro-protamine treatment. An unfavorable effect was observed on HDL-C, triglycerides, and VLDL-C. Combinations with more insulin showed better glycemic control, and those with more V1 showed better lipid management (Table 2).

**Table 2:** Carbohydrate and lipids metabolic response, and vanadium biodistribution after three days of treatment

Metabolite (mg/dL)	NT-T1D (*n* = 5)	Lispro insulin (*n* = 5)	Ins-V1 (9:1) (*n* = 5)	Ins-V1 (7:3) (*n* = 5)	Ins-V1 (5:5) (*n* = 5)	Ins-V1 (3:7) (*n* = 5)	Ins-V1 (1:9) (*n* = 5)	NH_4_VO_3_ (*n* = 5)	Ins-DV10 (9:1) (*n* = 5)	Ins- DV10 (7:3) (*n* = 5)	Ins-DV10 (5:5) (*n* = 5)	Ins-DV10 (3:7) (*n* = 5)	Ins-DV10 (1:9) (*n* = 5)	Decavanadate (DV10) (*n* = 5)
Triglycerides	86 ± 4.5	124 ± 16▲	114 ± 12	120 ± 20	114 ± 28	110 ± 21	103 ± 16	118 ± 18	93 ± 9 **↓**	85 ± 8 **↓**	80 ± 7 **↓**	65 ± 4 ▼**↓**	78 ± 9 **↓**	75 ± 8 **↓**
Total cholesterol	183 ± 11	140 ± 8▼	138 ± 9 ▼	141 ± 9▼	145 ± 10▼	144 ± 9▼	150 ± 10	163 ± 13	120 ± 14▼	116 ± 9 ▼**↓**	110 ± 7 ▼**↓**	100 ± 4 ▼**↓**	115 ± 6 ▼**↓**	121 ± 8 ▼**↓**
VLDL-C	17.2 ± 0.5	24.2 ± 3.2▲	22.8 ± 5.4	24 ± 4▲	22.8 ± 5.2	22 ± 5.2	20.6 ± 3.2	23.6 ± 3.3	18.6 ± 1.8 **↓**	17 ± 1.6 **↓**	16 ± 1.4 **↓**	13 ± 0.8 ▼**↓**	15.6 ± 1.8 **↓**	15 ± 1.6 **↓**
LDL-C	80 ± 4.6	39.5 ± 4.3▼	45.1 ± 3.9▼	43.8 ± 5.2▼	53.9 ± 5.3 ▼↑	56.2 ± 4.8 ▼↑	54.8 ± 3.7 ▼↑	57.9 ± 5.9 ▼↑	27.1 ± 2.8 ▼**↓**	27.3 ± 3.6 ▼**↓**	28.3 ± 4.1 ▼**↓**	26.9 ± 3.7 ▼**↓**	31 ± 5.3▼	42.8 ± 5.9▼
HDL-C	85.8 ± 3.8	75.7 ± 2.7▼	70.5 ± 4.5▼	73.2 ± 4.1▼	68.3 ± 3.3 ▼**↓**	65.8 ± 4.2▼**↓**	74.6 ± 2.8▼	81.5 ± 4.9	74.3 ± 3.3▼	71.7 ± 4.2 ▼	65.7 ± 3.5 ▼**↓**	60.1 ± 2.9 ▼**↓**	68.4 ± 4.8	63.2 ± 3.7 ▼**↓**
FFA	12.1 ± 2.8	6.4 ± 1.4▼	6.1 ± 3.2▼	6.5 ± 1.8▼	6.8 ± 3.9▼	6.2 ± 2.6▼	7.1 ± 2.9▼	10.2 ± 3.3	5.8 ± 1.6▼	5.5 ± 1.1▼	4.6 ± 1.3▼	4.1 ± 0.9▼	4.7 ± 1.5▼	4.9 ± 1.6▼
Glucose	416 ± 14.5	126 ± 3.3▼	137 ± 7.5▼	175 ± 4.9 ▼↑	237 ± 6.7 ▼↑	252 ± 4.7 ▼↑	265 ± 9.5 ▼↑	338 ± 10.1 ▼↑	134 ± 4.5▼	132 ± 6.2▼	120 ± 6.4▼	122 ± 7.3▼	164 ± 4.7▼ ↑	212 ± 10.1 ▼↑
Fructosamine (mmol/L)	86 ± 7.2	65 ± 2.5▼	68.2 ± 3.3▼	69.2 ± 4.6 ▼	73.2 ± 3.1 ↑	75.4 ± 5.1 ↑	74.2 ± 4.8 ↑	78 ± 5.3 ↑	64 ± 2.3▼	65.3 ± 3.4▼	70 ± 4.1	68.5 ± 3.6▼	75.9 ± 3.8 ↑	77.3 ± 3.3 ↑
Vanadium														
Serum (µg/L)	ND	ND	0.0063± 0.0002 ▲↑	0.021± 0.009 ▲↑	0.038± 0.011 ▲↑	0.056± 0.008 ▲↑	0.078± 0.01 ▲↑	0.09± 0.015 ▲↑	0.008± 0.0004 ▲↑	0.028± 0.002 ▲↑	0.05± 0.005 ▲↑	0.075± 0.007 ▲↑	0.1± 0.04 ▲↑	0.12 ± 0.03 ▲↑
Liver (µg/g of tissue)	0.0002± 0.00001	0.0001± 0.00001▼	0.27± 0.05 ▲↑	0.092± 0.013 ▲↑	1.64± 0.21 ▲↑	2.44± 0.18 ▲↑	3.37± 0.22 ▲↑	3.9 ± 0.5 ▲↑	0.38± 0.08 ▲↑	1.27± 0.09 ▲↑	2.27± 0.09 ▲↑	3.38± 0.21 ▲↑	4.67± 0.17 ▲↑	5.4 ± 0.61 ▲↑
Muscle (µg/g of tissue)	0.00006± 0.000003	0.00003± 0.000002▼	0.21± 0.03 ▲↑	0.71± 0.09 ▲↑	1.26± 0.16 ▲↑	1.88± 0.33 ▲↑	2.6± 0.32 ▲↑	3.0 ± 0.7 ▲↑	0.28± 0.04 ▲↑	0.95± 0.02 ▲↑	1.7± 0.12 ▲↑	2.53± 0.14 ▲↑	3.5± 0.24 ▲↑	4.05 ± 0.19 ▲↑
Heart (µg/g of tissue)	0.00005± 0.000002	0.00002± 0.000001▼	0.13± 0.02 ▲↑	0.42± 0.08 ▲↑	0.76± 0.09 ▲↑	1.13± 0.11 ▲↑	1.56± 0.17 ▲↑	1.8 ± 0.3 ▲↑	0.18± 0.09 ▲↑	0.59± 0.09 ▲↑	1.07± 0.1 ▲↑	1.59± 0.09 ▲↑	2.21± 0.14 ▲↑	2.55 ± 0.28 ▲↑
Renal cortex (µg/g of tissue)	0.0003± 0.00001	0.0001± 0.00001▼	0.34± 0.04 ▲↑	1.13± 0.13 ▲↑	2.02± 0.14 ▲↑	3.0± 0.5 ▲↑	4.15± 0.13 ▲↑	4.8 ± 0.7 ▲↑	0.38± 0.04 ▲↑	1.29± 0.11 ▲↑	2.3± 0.08 ▲↑	3.43± 0.37 ▲↑	4.75± 0.09 ▲↑	5.49 ± 0.44 ▲↑
Renal medulla (µg/g of tissue)	0.0001± 0.00001	0.0001± 0.00001	0.11± 0.02 ▲↑	0.35± 0.06 ▲↑	0.63± 0.08 ▲↑	0.94± 0.16 ▲↑	1.3± 0.16 ▲↑	1.5 ± 0.3 ▲↑	0.18± 0.05 ▲↑	0.62± 0.05 ▲↑	1.1± 0.09 ▲↑	1.65± 0.25 ▲↑	2.28± 0.13 ▲↑	2.64 ± 0.26 ▲↑

The results shown are the average of 5 separate experimental animals ± SEM. (▲/▼) Indicates a signiﬁcant difference from the NT-T1D group. (↓/↑), Signiﬁcant difference from Lispro insulin group. P ≤ 0.05 by one-way ANOVA with Dunnet post hoc test

Meanwhile, ins-DV10 compounds performed better in lipid and carbohydrate management, improving dyslipidemia and achieving glycemia levels close to physiological levels. Decavanadate treatment reduced total cholesterol (33.9% and 13.6%) and HDL (26.3% and 16.5%) compared with the NT-DT1 and Lispro-insulin groups, respectively; LDL-C (46.8%), FFA (62%), and glucose (49%) compared with the NT-DT1 group, but these were not better than lispro-protamine treatment; triglyceride, HDL-C, and fructosamine did not improve regarding the NT-DT1 group, even worsening than lispro-protamine treatment. Equimolar combinations of ins-DV10 1:9 to 9:1 improved glycemia by 60.6% to 67.8%, total cholesterol by 37.2% to 34.4%, LDL-C by 61.3% to 66.1%, FFA by 61.2% to 52.1%, and fructosamine by 20.3% to 25.6%, showing better or equal effectiveness than Lispro-protamine treatment. All ins-DV10 combinations prevent hypertriglyceridemia and VLDL-C better than Lispro-protamine treatment and diminish HDL-C concentrations (Table 2).

At the same time, vanadium distribution and accumulation were evaluated in tissues that use insulin to manage carbohydrate or lipid metabolism. The distribution was analyzed in the serum on the third day of treatment. The results showed that vanadium increased in proportion to the administered dose (equimolar combinations). The NH_4_VO_3_ group had vanadium serum concentrations that ranged from 0.0063 to 0.09 µg/L, with vanadium levels proportional to the molar ratio of insulin administered. Tissues also showed the same behavior. Vanadium accumulation was highest in the renal cortex (4.8 µg/g of tissue), followed by the liver (3.9 µg/g of tissue), muscle (3.0 µg/g of tissue), heart (1.8 µg/g of tissue), and renal medulla (1.5 µg/g of tissue). Serum vanadium concentration after DV10 treatment was 0.12 µg/L, while ins-DV10 combinations were from 0.1 µg/L (1:9) to 0.008 µg/L (9:1). In the tissues, the renal cortex demonstrated the most significant vanadium accumulation (5.49 µg/g of tissue), followed by the liver (5.4 µg/g of tissue), muscle (4.05 µg/g of tissue), renal medulla (2.64 µg/g of tissue), and heart (2.55 µg/g of tissue). All of them in the DV10 group, which was higher than vanadium accumulates in the NH_4_VO_3_ group and ins-DV10 combinations (Table 2).

**Fig. 3:**
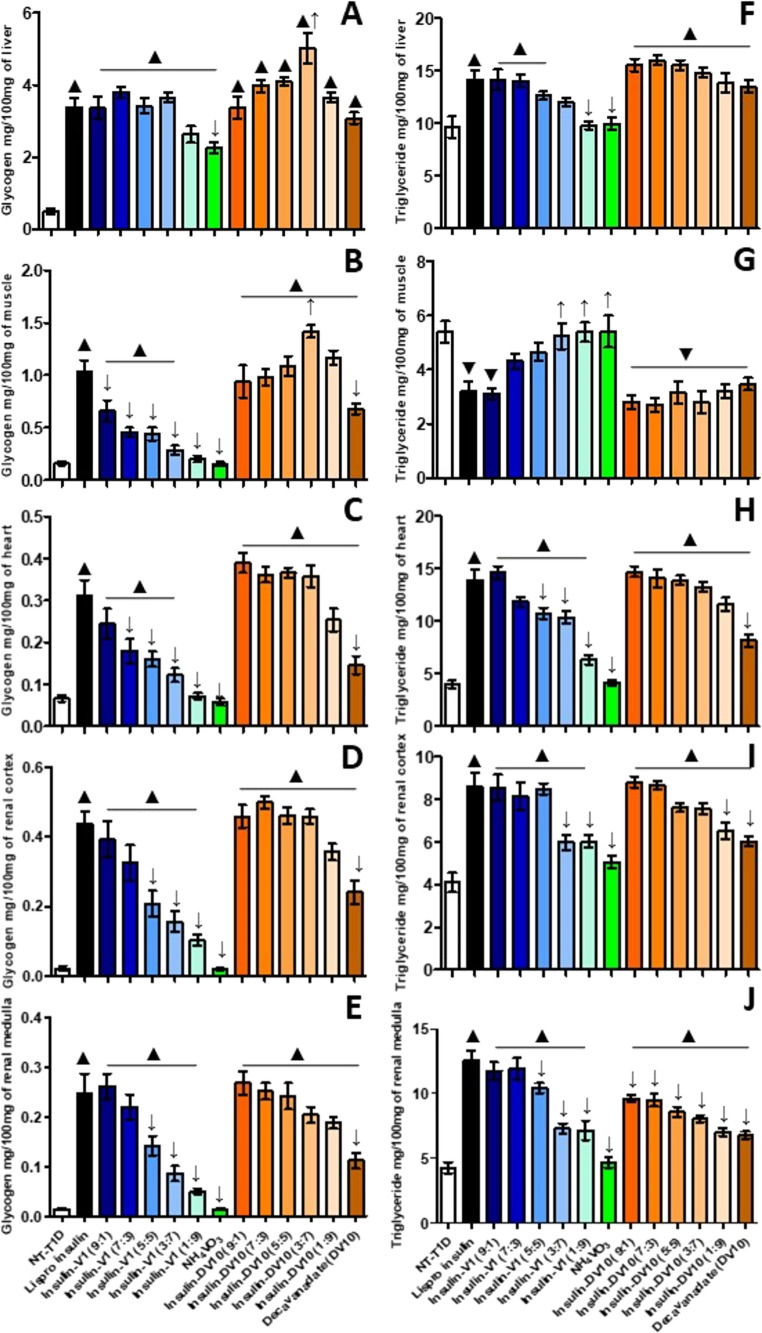
Glycogen and triglyceride storage after acute insulin-functionalized administration. **(A)** Liver glycogen concentration. **(B)** Muscle glycogen concentration. **(C)** Heart glycogen concentration. **(D)** Renal cortex glycogen concentration. **(E)** Renal medulla glycogen concentration. **(F)** Liver triglyceride concentration. **(G)** Muscle triglyceride concentration. **(H)** Heart triglyceride concentration. **(I)** Renal cortex triglyceride concentration. **(J)** Renal medulla triglyceride concentration. The results shown are the average of 5 separate experimental animals ± SEM. (▲/▼) Indicates a significant difference from the Lispro insulin group. *P* ≤ 0.05 by one-way ANOVA with Dunnett post hoc test

In tissues, final actions linked to insulin signaling, such as glycogen and triglyceride synthesis and storage, were evaluated. In the liver, glycogen content significatively increased with all treatments (2.1–3.9 mg/100 mg of tissue). Ins-V1 combinations showed glycogen content similar to Lispro treatment (3.7–3.9 mg/100 mg of tissue), except for the NH_4_VO_3_ dose (2.1 mg/100 mg of tissue). Ins-DV10 treatments improved glycogen synthesis and storage even more than Lispro treatment (3.8–5.0 mg/100 mg of tissue). DV10 treatment without insulin had a similar glycogen concentration to that of the Lispro insulin group (3.8 mg/100 mg of tissue; Fig. 3A). Hepatic triglyceride content also increased after Lispro treatment (from 9.5 to 14 mg/100 mg of tissue). Ins-V1 combination diminished hepatic triglyceride content proportionally to vanadium concentration, from 14 mg/100 mg of tissue (ins-V1 9:1) to 9.5 mg/100 mg of tissue in ins-V1 1:9 and NH_4_VO_3_ groups; likewise, ins-DV10 combinations diminished slightly triglyceride content from 15.5 mg/100 mg of tissue (ins-DV10 9:1) to 13.8 mg/100 mg of tissue (ins-DV10 1:9; Fig. 3F).

In the muscle and heart, Lispro treatment increased glycogen content (0.31 and 0.43 mg/100 mg of tissue). Ins-V1 combination improved glycogen content proportionally to insulin concentration, from 0.12 mg/100 mg of tissue (NH_4_VO_3_) to 0.6 mg/100 mg of tissue in ins-V1 9:1 (in muscle) and from 0.07 mg/100 mg of tissue (NH_4_VO_3_) to 0.25 mg/100 mg of tissue in ins-V1 9:1 (in heart). Meanwhile, DV10 alone increased glycogen content by 480% and 200% in muscle and heart, respectively. Ins-DV10 combinations showed to be better than Lispro treatment to generate glycogen proportionally to DV10 in muscle (until 1.4 mg/100 mg of tissue; ins-DV10 3:7) and to insulin content in heart, from 0.27 mg/100 mg of tissue (ins-DV10 1:9) to 0.39 mg/100 mg of tissue (ins-DV10 9:1; Fig. 3B and C). In the muscle, triglyceride content decreased by 43.9% and 47.1% after Lispro and ins-V1 (9:1) treatment, respectively. Other ins-V1 combinations were not different from the NT-DT1 group; however, they showed a trend to diminish when containing more insulin (Fig. 3G). Treatments with DV10 alone and in combination with insulin showed a muscular triglyceride content similar to that with Lispro (≈ 3 mg/100 mg of tissue). In the heart, the triglyceride content was from 8.1 mg/100 mg of tissue (DV10 group) to 14.7 mg/100 mg of tissue (ins-DV10 9:1 group; Fig. 3H).

In the renal cortex and medulla, Lispro treatment increased glycogen content to 0.44 mg and 0.25 mg/100 mg of tissue, respectively. NH_4_VO_3_ administration did not ameliorate glycogen content in either renal region, at 0.02 mg and 0.014 mg/100 mg of tissue, respectively, values close to those of the NT-DT1 group. However, ins-V1 combinations increase glycogen content from 0.1 mg (cortex) and 0.05 mg/100 mg of tissue (medulla) in the ins-V1 1:9 group to 0.4 mg (cortex) and 0.26 mg/100 mg of tissue (medulla) in the ins-V1 9:1 group. Treatments with DV10 alone and in combination with insulin improved glycogen content in both renal regions from 0.24 mg (cortex) and 0.11 mg/100 mg of tissue (medulla) in the DV10 group to 0.5 mg (cortex) and 0.27 mg/100 mg of tissue (medulla) in the ins-DV10 9:1 group (Fig. 3D and E). Meanwhile, triglyceride content increased with Lispro treatment, reaching 8.6 mg in the renal cortex and 12.6 mg/100 mg of tissue in the renal medulla. In the renal cortex, NH_4_VO_3_ administration increased triglyceride concentration by 36% and this effect persisted with the ins-V1 combinations, reaching 108.8% in the ins-V1 9:1 treatment, similarly to Lispro treatment. In the renal medulla, NH_4_VO_3_ treatment did not affect triglyceride concentration, but ins-V1 combinations progressively increased triglyceride levels from 67% (ins-V1 1:9) to 174% (ins-V1 9:1), as with Lispro treatment. In the renal cortex, NH_4_VO_3_ administration increased triglyceride concentration by 36% and this effect persisted with the ins-V1 combinations, reaching 108.8% in the ins-V1 9:1 treatment, similarly to Lispro treatment. In the renal cortex and medulla, DV10 treatment increased triglyceride concentration by 47% and 58.4%, respectively. In comparison, the treatment with ins-DV10 combinations continued to increase it from 59.5% (ins-DV10 1:9) to 115% (ins-DV10 9:1) in renal cortex, and from 64% (ins-DV10 1:9) to 124.8% (ins-DV10 9:1) in renal medulla (Fig. 3I and J).

**Fig. 4:**
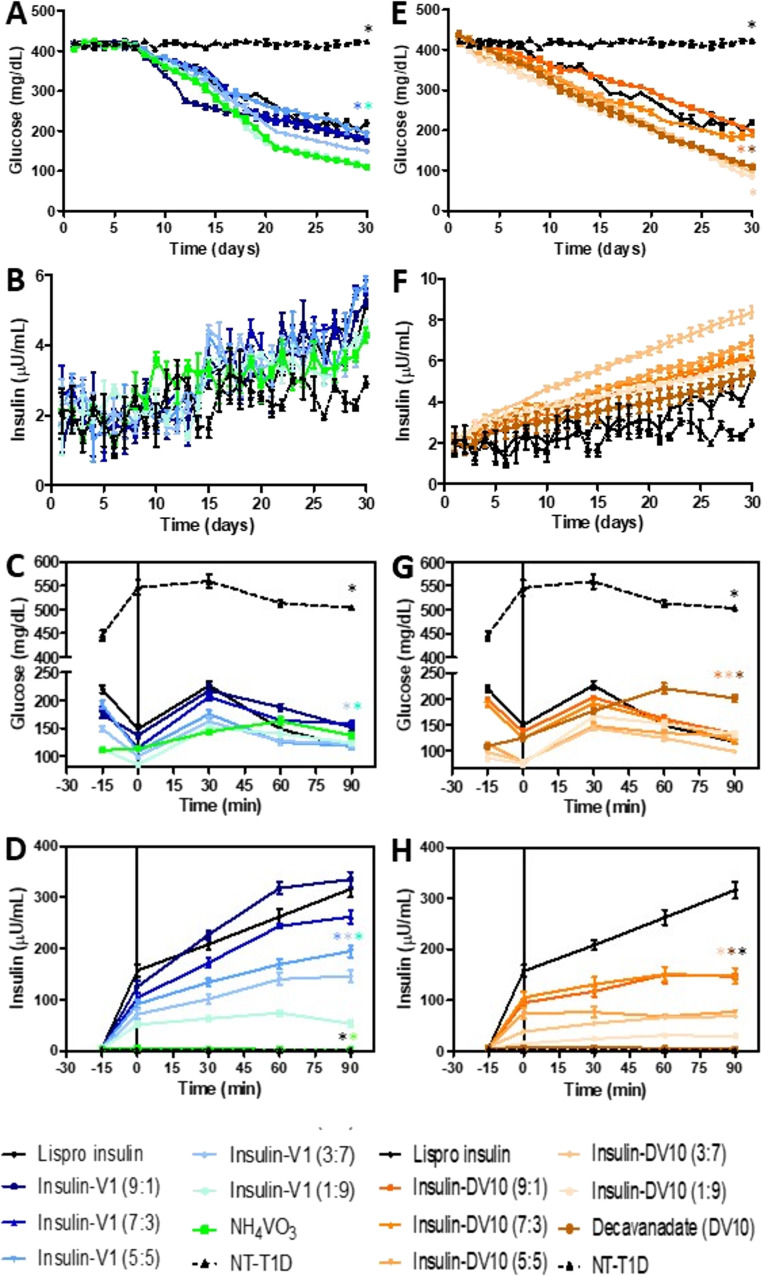
Fasting glucose and insulin, pharmacodynamics, and pharmacokinetics after oral glucose tolerance test in rats treated with insulin-functionalized in the long term. **(A)** Fasting glucose in insulin-vanadate groups. **(B)** Fasting insulin in insulin-vanadate groups. **(C)** Pharmacodynamics of insulin-vanadate groups after OGTT. **(D)** Pharmacokinetics of insulin-vanadate groups after OGTT. **(E)** Fasting glucose in insulin-decavanadate groups. **(F)** Fasting insulin in insulin-decavanadate groups. **(G)** Pharmacodynamics of insulin-decavanadate groups after OGTT. **(H)** Pharmacokinetics of insulin-decavanadate groups after OGTT. The results shown are the average of 5 separate experimental animals ± SEM. (*) Indicates a significant difference from the Lispro insulin group. *P* ≤ 0.05 by two-way Repeated Measures ANOVA was conducted to evaluate treatment effects, followed by Tukey’s post hoc test

The cumulative effect of Lispro-protamine insulin was observed, and its activity persisted, as evidenced by daily fasting glycemia, which decreased from 400 mg/dL to 219.7 mg/dL before treatment administration. However, ins-V1 combinations demonstrated better performance than Lispro-protamine treatment (F_(7,203)_ = 44.73; *p* < 0.001). The NH_4_VO_3_ treatment reduced fasting glucose by an average of 112–148.7.7 mg/dL (*p* = 0.015). The ins-V1 9:1, 7:3, and 3:7 formulations showed no significant differences compared to Lispro-protamine treatment (*p* > 0.05), demonstrating that they maintain similar efficacy to commercial insulin in the long term. The 5:5 and 1:9 ratios showed significant differences compared to Lispro (*p* < 0.05). Specifically, the (5:5) ratio resulted in the lowest average glucose levels of all the tested mixtures (Fig. 4A). Analysis of variance showed significant effects for treatment, time, and their interaction (F_(7,203)_ = 1.50; *p* < 0.001). However, the magnitude of the difference was smaller than in the glucose tests, consistent with the nature of daily insulin dosing. The significant interaction (*p* < 0.001) indicates that insulin levels do not remain constant and vary with treatment throughout the study month. No statistically significant differences were found between any of the ins-V1 formulations (9:1, 7:3, 5:5, 3:7, 1:9) and commercially available Lispro Insulin (*p* > 0.05). This suggests that all mixtures maintain plasma insulin levels similar to those of standard therapy over a prolonged period (Fig. 4B).

In the same way, all insulin decavanadate combinations reduced fasting glycemia at the 30-day analysis to below 200 mg/dL (F_(7,203)_ = 34.55; *p* < 0.001). The significant interaction (*p* < 0.001) indicates that glycemic profiles over the 30 days are directly dependent on the administered formulation. As expected, commercial insulin maintained significantly lower glucose levels than the diabetic control (NT-T1D, *p* < 0.001). Furthermore, it showed levels that were considerably higher than those with decavanadate alone (DV10, *p* = 0.0005), suggesting that DV10 alone has a potent overall hypoglycemic effect in this model (112 mg/dL). The ins-DV 9:1 and 7:3 formulations showed no statistically significant differences with Lispro-protamine activity (*p* > 0.05), indicating similar efficacy. The formulations with a higher proportion of decavanadate (5:5, 3:7, 1:9) showed significantly lower glucose levels than the commercial treatment (*p* < 0.001), ins-DV10 5:5 (110 mg/dL), and ins-DV10 3:7 and 1:9 (85 mg/dL), suggesting a more potent or longer-lasting effect at these ratios (Fig. 4E).

Meanwhile, analysis of variance showed statistically significant effects, highlighting the impact of different formulations on plasma insulin stability (F_(7,203)_ = 2.54; *p* < 0.001). The significant interaction (*p* < 0.001) confirms that plasma insulin behavior throughout the month differs according to the administered ins-DV10 mixture ratio. In this 30-day experiment, Lispro-protamine treatment did not show a significant difference from diabetic control (NT-T1D; *p* = 0.062) in cumulative basal levels. However, it was significantly different from decavanadate alone (DV10, *p* = 0.007). All Insulin-DV formulations (9:1, 7:3, 5:5, 3:7, and 1:9) showed significantly higher insulin levels than commercial insulin (*p* < 0.001). This suggests that the complex with decavanadate substantially improves the retention of insulin in plasma compared to insulin alone. The insulin-to-decavanadate ratio 3:7 showed the greatest increase in plasma insulin, significantly higher than all other ratios tested (*p* < 0.001). The hormone content in the ins-DV10 1:9 formulation was detectable and accumulated in plasma in the long term, demonstrating that even a small amount of insulin added to the complex is detectable and effective (Fig. 4F).

We also analyzed glucose tolerance 15 min after each treatment. Glycemia in groups administered with ins-V1 combinations was between 80 and 150 mg/dL at 0 min; at 30 min after glucose load, glycemia oscillated from 140 to 215 mg/dL; at 60 min, it was between 130 and 190 mg/dL, and at 90 min, it was 110–150 mg/dL. The analysis demonstrated that the speed and magnitude of the response to the glucose load vary substantially among the different treatment groups (F_(7,28)_ = 16.41; *p* < 0.001). There were no significant differences between commercial insulin and the ins-V1 9:1, 7:3, and 5:5 formulations (*p* > 0.05), indicating that these mixtures have hypoglycemic efficacy similar to commercial insulin during an oral glucose load. However, the formulations with lower insulin content (3:7 and 1:9) resulted in significantly lower glucose levels than Lispro-protamine insulin alone (*p* < 0.05), suggesting a synergistic or prolonged effect due to the vanadate or its oligomers (tetravanadate). NH_4_VO_3_ alone showed remarkable efficacy, being significantly different from Lispro treatment (*p* = 0.045) in maintaining lower glucose levels on average throughout the test (Fig. 4C). The analysis of insulin response to OGTT revealed highly significant effects for both treatment and time, as well as a significant interaction (F_(7,28)_ = 45.87; *p* < 0.001). Plasma insulin levels respond differently over time depending on the insulin-V1 mixture used. The ins-V1 9:1, 7:3, and 5:5 formulations showed no statistically significant differences from commercially available insulin (*p* > 0.05), indicating comparable insulin levels. In contrast, the formulations with lower insulin content (3:7 and 1:9) showed significantly lower insulin levels than Lispro treatment (*p* < 0.05), consistent with the lower hormone dose administered in those mixtures (Fig. 4D).

A similar effect was observed in animals treated with ins-DV10 and decavanadate, glucose concentration was 75–136 mg/dL at 0 min; at 30 min after glucose load, glycemia oscillated from 143 to 202 mg/dL; at 60 min, it was between 124 and 220 mg/dL, and at 90 min, it was 98–201 mg/dL (Fig. 4G). The ins-DV10 3:7 combination showed the best performance, even better than Lispro-protamine treatment. The highly significant interaction (F_(7,28)_ = 23.31; *p* < 0.001) indicates that the different insulin-DV10 formulations modify glucose absorption or regulation dynamics differently throughout the 90-minute test. No significant differences were found between commercial insulin and the ins-DV10 9:1 and 7:3 formulations (*p* > 0.9), indicating equivalent hypoglycemic efficacy. However, the ins-DV10 5:5, 3:7, and 1:9 combinations showed significantly lower glucose levels than Lispro insulin treatment alone (*p* < 0.01). This suggests that increasing the proportion of decavanadate in the complex enhances glycemic control during an oral glucose load. Decavanadate showed similar efficacy to commercial insulin (*p* = 0.999) and the 9:1 mixture, but was outperformed by mixtures with higher vanadate content, such as the 3:7 mixture (*p* = 0.001). The dynamics of insulin release or plasma levels vary significantly depending on the ins-DV10 formulation used and the time elapsed after the glucose load (F_(7,28)_ = 30.75; *p* < 0.001). Commercial insulin showed significantly higher insulin levels than all ins-DV10 mixtures (*p* < 0.001). Among the mixtures, the 9:1 and 7:3 formulations showed significantly higher insulin levels than mixtures with higher DV10 content (such as 1:9). There were no significant differences between the untreated group (NT-T1D) and the decavanadate-only group (*p* = 1.0), which was expected since neither of these groups received exogenous insulin. The ins-DV10 1:9 and 3:7 groups also showed no significant differences compared to the diabetic control (*p* > 0.05), reflecting the low insulin dose in these ratios. Therefore, serum insulin disposition was greater with ins-V1 treatments (335 µU/mL) than with ins-DV10 treatments (146.7 µU/mL), suggesting that the hormone in the ins-DV10 compounds is slowly released (Fig. 4D and H).

After 1 month, a fasting serum panel of carbohydrates and lipids was analyzed. Lispro-protamine treatment reduced glucose (48%), fructosamine (33.7%), total cholesterol (16.4%), LDL-C (35.4%), FFA (77.3%), and TG (63.2%) compared with the NT-T1D group. NH_4_VO_3_ treatment improved glucose, fructosamine, TG, total cholesterol, LDL-C, and FFA levels by 73.8%, 25.4%, 58.4%, 34%, 61%, and 48.7% respectively, compared with the NT-T1D group; likewise, glucose and VLDL-C concentrations were better than the Lispro-protamine group, but not FFA and fructosamine concentrations. Equimolar combinations of ins-V1 1:9 to 9:1 improved glycemia by 58.9% to 74% compared with the NT-T1D group and 16.6% to 47.3% compared with the Lispro-protamine treatment. Fructosamine and TG decreased by 35.6% to 41.9% and 55.8% to 70.1%, respectively, compared with the NT-T1D group, but did not differ from Lispro-protamine treatment. Total cholesterol decreased by 24.8% to 51.9% compared with the NT-T1D group; the ins-V1 1:9 resulted in the best treatment, even than the Lispro-protamine group (42.5%). The ins-V1 1:9 treatment also diminished VLDL-C concentration below the NT-T1D group and Lispro-protamine treatment by 60.2% and 63.4%, respectively. The ins-V1 combinations 9:1, 3:7, and 1:9 diminished LDL-C concentration by 49.1%, 43.3%, and 78.9% compared with the NT-T1D group; the treatment with ins-V1 1:9, again, was better than Lispro by 66.7%. All ins-V1 combinations decreased HDL-C concentration by 15.5% to 27.6% compared with the NT-T1D group, but only 5:5, 7:3, and 9:1 combinations were better than Lispro-protamine treatment. Besides, all ins-V1 combinations decreased FFA concentration by 57.8% to 69.5%, demonstrating performance similar to Lispro-protamine treatment (Table 3).

**Table 3:** Carbohydrate and lipids metabolic response and vanadium accumulation after one month of treatment

Metabolite (mg/dL)	NT-T1D (*n* = 5)	Lispro insulin (*n* = 5)	Ins-V1 (9:1) (*n* = 5)	Ins-V1 (7:3) (*n* = 5)	Ins-V1 (5:5) (*n* = 5)	Ins-V1 (3:7) (*n* = 5)	Ins-V1 (1:9) (*n* = 5)	NH_4_VO_3_ (*n* = 5)	Ins-DV10 (9:1) (*n* = 5)	Ins- DV10 (7:3) (*n* = 5)	Ins- DV10 (5:5) (*n* = 5)	Ins- DV10 (3:7) (*n* = 5)	Ins- DV10 (1:9) (*n* = 5)	Decavanadate (DV10) (*n* = 5)
Triglycerides	231 ± 11	85 ± 21▼	102 ± 34▼	74 ± 23▼	69 ± 14▼	74 ± 21▼	71 ± 16▼	96 ± 26▼	97 ± 6▼	108 ± 14▼	67 ± 14▼	57 ± 3 **↓**▼	61 ± 7▼	128 ± 33▼
Total cholesterol	149 ± 5	124.6 ± 22	101 ± 6.7 ▼	133.8 ± 9	112.4 ± 1.2▼	101.2 ± 16▼	71.6 ± 9 **↓**▼	98.3 ± 12▼	132.6 ± 8	144 ± 9.5	112 ± 8.6▼	127 ± 13.5	97.5 ± 2▼	149 ± 9
VLDL-C	9.3 ± 2.8	10.1 ± 1.9	7.3 ± 1.0	14.7 ± 0.8 ↑▲	12.5 ± 1.3	9.15 ± 1.2	3.7 ± 0.4 **↓**▼	5.9 ± 0.9 **↓**	11.8 ± 3.1	14.1 ± 2.3	9.6 ± 1.4	16.3 ± 1.4 ↑▲	8.5 ± 1.0	13.6 ± 1.5
LDL-C	64.8 ± 6.5	41.2 ± 9.4▼	33 ± 3.2▼	55.8 ± 5.8	44.6 ± 8.5	36.8 ± 9.2▼	13.7 ± 3.6 **↓**▼	25.3 ± 4.1 **↓**▼	51.8 ± 4	64.1 ± 4.5 ↑	35.4 ± 3.5▼	53.7 ± 7.3	28 ± 2.8▼	69.4 ± 8 ↑
HDL-C	74.9 ± 2.9	73.3 ± 11.5	60.7 ± 5.5▼	63.3 ± 1.5▼	55.3 ± 3.5 **↓**▼	57.3 ± 3.0 **↓**▼	54.2 ± 5.8 **↓**▼	67.1 ± 3.9	69 ± 6.5	65 ± 11	67 ± 11.5	57 ± 2.5 **↓**▼	61 ± 6.7	66 ± 3.0
FFA	15.4 ± 2.8	3.5 ± 0.8▼	4.7 ± 1.1▼	5.1 ± 0.9▼	5.3 ± 1.0▼	5.9 ± 1.5▼	6.5 ± 1.1 ↑▼	7.7 ± 1.8 ↑▼	3.6 ± 1.2▼	3.8 ± 1.1▼	3.4 ± 0.9▼	2.9 ± 0.6▼	3.3 ± 0.8▼	3.6 ± 0.7▼
Glucose	423 ± 22.1	220 ± 10.4▼	174.3 ± 8.8 **↓**▼	185 ± 6.4 **↓**▼	192 ± 7.4 **↓**▼	148 ± 5.5 **↓**▼	110 ± 4.3 **↓**▼	111 ± 10.1 **↓**▼	199 ± 6▼	189 ± 8 **↓**▼	112 ± 7 **↓**▼	96 ± 4 **↓**▼	85 ± 9 **↓**▼	108 ± 7 ▼
Fructosamine (mmol/L)	315 ± 14.7	209 ± 11.9▼	203 ± 9.4▼	200 ± 7.3▼	193 ± 8.1▼	183 ± 8.8 ▼	199 ± 10.4▼	235 ± 6.2 ↑▼	175 ± 4.4 **↓**▼	160 ± 5.1 **↓**▼	141 ± 8.2 **↓**▼	120 ± 4.8 **↓**▼	125 ± 4.2 **↓**▼	128 ± 5.4 **↓**▼
Vanadium														
Serum (µg/L)	ND	ND	0.032± 0.008 ↑▲	0.1 ± 0.07 ↑▲	0.19± 0.02 ↑▲	0.28± 0.06 ↑▲	0.39± 0.16 ↑▲	0.45± 0.11 ↑▲	0.04± 0.004 ↑▲	0.14± 0.04 ↑▲	0.23± 0.09 ↑▲	0.32± 0.04 ↑▲	0.41± 0.08 ↑▲	0.47 ± 0.11 ↑▲
Liver (µg/g of tissue)	0.0001± 0.00005	0.00001± 0.000003▼	2.1 ± 0.6 ↑▲	7.05 ± 0.8 ↑▲	12.6 ± 0.5 ↑▲	18.8 ± 1.2 ↑▲	26 ± 2.3 ↑▲	30 ± 3.3 ↑▲	2.5 ± 0.7 ↑▲	7.9 ± 0.6 ↑▲	13.2 ± 1.4 ↑▲	18.5 ± 2.3 ↑▲	23.3 ± 3.9 ↑▲	26.9 ± 4.5 ↑▲
Muscle (µg/g of tissue)	0.00003± 0.000001	0.00001± 0.000001▼	1.9 ± 0.7 ↑▲	6.3 ± 0.6 ↑▲	11.3 ± 0.4 ↑▲	16.9 ± 1.1 ↑▲	23.4 ± 2.5 ↑▲	27 ± 2.4 ↑▲	2.0 ± 0.5 ↑▲	6.4 ± 0.8 ↑▲	10.7 ± 1.1 ↑▲	15 ± 1.7 ↑▲	18.9 ± 2.6 ↑▲	21.8 ± 5.7 ↑▲
Heart (µg/g of tissue)	0.00002± 0.000001	0.00001± 0.000001▼	1.0 ± 0.3 ↑▲	3.4 ± 0.4 ↑▲	6.0 ± 0.5 ↑▲	9 ± 0.85 ↑▲	12.5 ± 1.9 ↑▲	14.4 ± 2.7 ↑▲	1.3 ± 0.3 ↑▲	3.9 ± 0.5 ↑▲	6.6 ± 0.8 ↑▲	9.2 ± 0.8 ↑▲	11.6 ± 1.7 ↑▲	13.4 ± 2.4 ↑▲
Renal cortex (µg/g of tissue)	0.0001± 0.00003	0.00002± 0.000003▼	3.8 ± 0.8 ↑▲	12.7 ± 1.2 ↑▲	22.7 ± 2.5 ↑▲	33.8 ± 4.1 ↑▲	46.7 ± 5.7 ↑▲	54 ± 8.3 ↑▲	2.7 ± 0.6 ↑▲	8.4 ± 1.3 ↑▲	14 ± 1.8 ↑▲	19.6 ± 3.1 ↑▲	24.7 ± 3.4 ↑▲	28.6 ± 4.8 ↑▲
Renal medulla (µg/g of tissue)	0.00005± 0.000004	0.00001± 0.000002▼	1.7 ± 0.2 ↑▲	5.6 ± 0.4 ↑▲	10.1 ± 1.3 ↑▲	15 ± 1.7 ↑▲	20.8 ± 2.9 ↑▲	24 ± 3.9 ↑▲	1.1 ± 0.05 ↑▲	3.5 ± 0.7 ↑▲	5.8 ± 0. 9 ↑▲	8.1 ± 1.3 ↑▲	10.2 ± 1.6 ↑▲	11.8 ± 1.9 ↑▲

The results shown are the average of 5 separate experimental animals ± SEM. (▼) Indicates a significant difference from the NT-T1D group. (**↓/↑**), Significant difference from Lispro insulin group. *P* ≤ 0.05 by one-way ANOVA with Dunnet post hoc test

Likewise, DV10 treatment improved glucose, fructosamine, TG, and FFA levels by 74.5%, 59.4%, 44.6%, and 76.6%, respectively, compared with the NT-T1D group. Ins-DV10 1:9 to 9:1 improved glycemia and fructosamine by 53% to 79.9% and by 44.4% to 60.3%, respectively, compared with the NT-T1D group; from 14.1% to 61.4% and 16.3% to 40.2%, respectively, compared with the Lispro-protamine treatment. Triglycerides and FFA decreased by 58% to 75.3% and by 75.3% to 81.2%, respectively, compared with the NT-T1D group, but only the ins-DV10 3:7 combination was superior to Lispro-protamine treatment in reducing TG. The ins-DV10 1:9 and 5:5 combinations reduced total cholesterol and LDL-C by 34.6% and 56.8%, and by 24.8% and 45.4%, respectively, compared with the NT-T1D group, but none improved these parameters more than Lispro-protamine treatment. HDL-C decreased only with the ins-DV10 3:7 combination by 23.8% compared with the NT-T1D group and by 22.2% compared with Lispro-protamine treatment (Table 3). Additionally, vanadium in serum significantly increased in the treated groups with NH_4_VO_3_ (0.45 µg/L) and ins-V1 combinations after 1 month, with higher concentrations in the groups with the higher vanadium dose, from 0.032 µg/L (ins-V1 9:1) to 0.39 µg/L (ins-V1 1:9). Similar results were observed in groups administered with DV10 and ins-DV10 combinations, 0.47 µg/L in DV10 group, and from 0.04 µg/L (ins-DV10 9:1) to 0.41 µg/L (ins-DV10 1:9). In the renal cortex vanadium concentration was from 3.8 to 54 µg/g of tissue (ins-V1 9:1 to NH_4_VO_3_) and from 2.7 to 28.6 µg/g of tissue (ins-DV10 9:1 to DV10). In the liver, it was from 2.1 to 30 µg/g of tissue (ins-V1 9:1 to NH_4_VO_3_) and from 2.5 to 26.9 µg/g of tissue (ins-DV10 9:1 to DV10). In the muscle, it was from 1.9 to 27 µg/g of tissue (ins-V1 9:1 to NH_4_VO_3_) and from 2.0 to 21.8 µg/g of tissue (ins-DV10 9:1 to DV10). In the renal medulla, it was from 1.7 to 24 µg/g of tissue (ins-V1 9:1 to NH_4_VO_3_) and from 1.1 to 11.8 µg/g of tissue (ins-DV10 9:1 to DV10). In the heart, it was from 1.0 to 14.4 µg/g of tissue (ins-V1 9:1 to NH_4_VO_3_) and from 1.3 to 13.4 µg/g of tissue (ins-DV10 9:1 to DV10; Table 3).

**Fig. 5:**
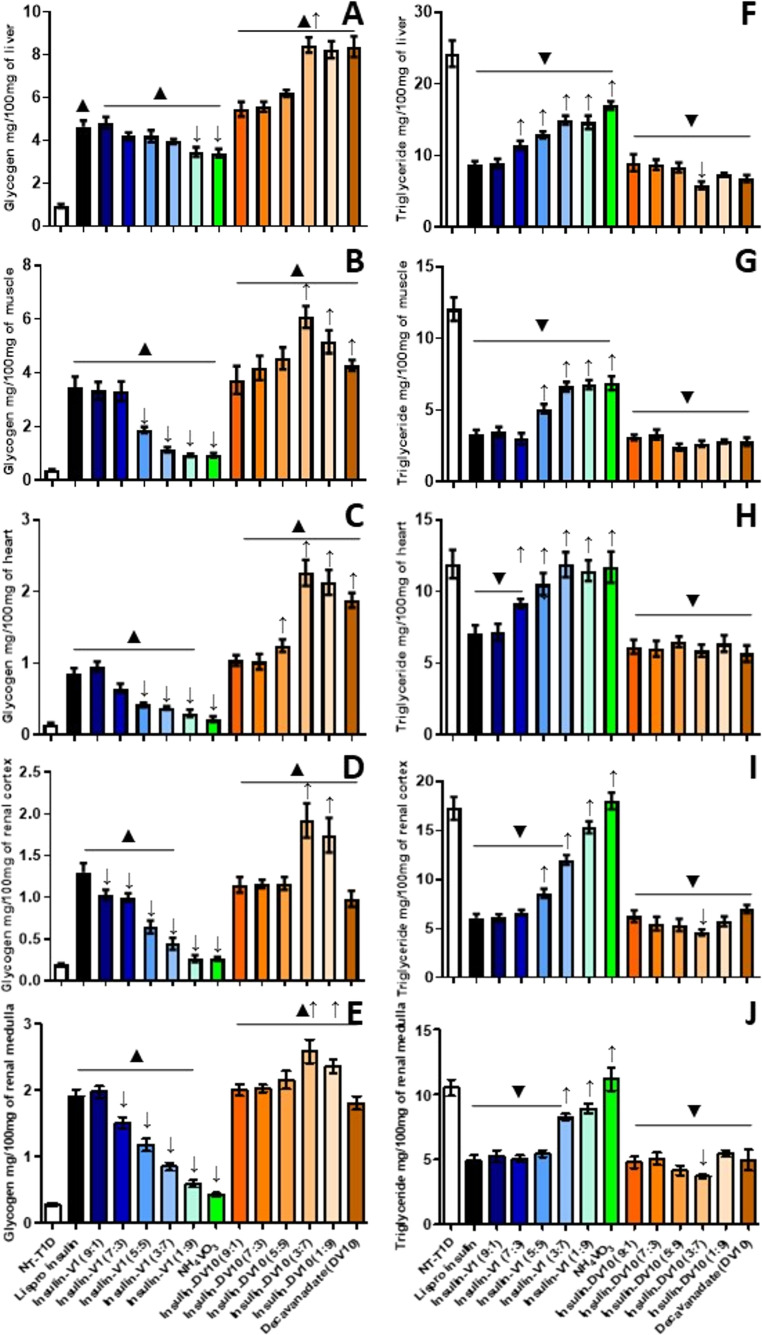
Glycogen and triglyceride storage after long-term insulin-functionalized administration. **(A)** Liver glycogen concentration. **(B)** Muscle glycogen concentration. **(C)** Heart glycogen concentration. **(D)** Renal cortex glycogen concentration. **(E)** Renal medulla glycogen concentration. **(F)** Liver triglyceride concentration. **(G)** Muscle triglyceride concentration. **(H)** Heart triglyceride concentration. **(I)** Renal cortex triglyceride concentration. **(J)** Renal medulla triglyceride concentration. The results shown are the average of 5 separate experimental animals ± SEM. (▲/▼) Indicates a significant difference from the Lispro insulin group. *P* ≤ 0.05 by one-way ANOVA with Dunnett post hoc test

After 1 month of Lispro-protamine administration, glycogen levels were greater than NT-DT1 in the liver (393%), muscle (787%), heart (473%), renal cortex (584%), and renal medulla (582%). The NH_4_VO_3_ treatment increased glycogen concentration only in the liver (261%), muscle (144%), and heart (104%). The ins-V1 combinations demonstrated tissue glycogen content augmented in relation to a higher insulin proportion compared with NT-DT1 in the liver (from 268% to 414%), muscle (from 142% to 765%), heart (from 104% to 542%), renal cortex (from 128% to 432%), and renal medulla (from 113% to 611%). Consistently, the ins-V1 combinations 9:1 and 7:3 (except in the renal cortex) showed performance similar to Lispro-protamine treatment. Conversely, DV10 and its combinations with insulin were superior to Lispro-protamine treatment. Glycogen level augmented after 1 month of DV10 treatment in the liver (791%), muscle (1008%), heart (1160%), renal cortex (407%), and renal medulla (552%). The ins-DV10 combinations augmented tissue glycogen content in relation to a higher DV10 proportion compared with NT-DT1 in the liver (from 480% to 776%), muscle (from 868% to 1475%), heart (from 597% to 1416%), renal cortex (from 492% to 889%), and renal medulla (from 621% to 830%). In fact, the ins-DV10 combinations 3:7 and 1:9 showed the best performance, even better than Lispro-protamine treatment (Fig. 5A-E).

Finally, tissue triglyceride accumulation was analyzed. In all analyzed tissues, Lispro-protamine treatment reduced it compared with NT-DT1: liver (63.8%), muscle (72.8%), heart (40.4%), renal cortex (64.8%), and renal medulla (53.6%), demonstrating insulin activity on lipid homeostasis and the prevention of steatosis. The NH_4_VO_3_ treatment had a positive effect only in the liver (29.7%) and muscle (42.9%). The ins-V1 9:1 combination showed the best performance, similar to Lispro-protamine treatment across all analyzed tissues, and ins-V1 7:3 decreased triglyceride content in muscle (75%), renal cortex (61.4%), and renal medulla (52.1%). DV10 treatment produced results similar to Lispro-protamine insulin, reducing triglyceride accumulation in the liver (71.8%), muscle (76.7%), heart (52.3%), renal cortex (59.4%), and renal medulla (52.9%). In general, all ins-DV10 combinations observed similar results to Lispro-protamine and DV10 treatments; however, the ins-DV10 3:7 combination showed better results than both, in the liver (75.8%), muscle (77.9%), heart (50.7%), renal cortex (73.2%), and renal medulla (64.8%; Fig. 5F-J).

## Discussion

### Characterization of Insulin Functionalized with Vanadium

As a first step, we modified HUMALOG^®^ insulin by removing zinc and other metals and then functionalized it with V1 or DV10 under different molar conditions. However, we chose to inform the molar combination (5:5) because molecular modifications were more tangible. For the first time, a possible influence from V1, independent of its speciation, on the electronic environment of the insulin molecules, attributable to charge transfer transitions of vanadium-oxygen. This interaction is crucial because it may help us understand how V1 could be used to improve insulin sensitivity and treat diabetes. Tyrosine and histidine residues in the insulin chains appear to be involved in this interaction. Both 240–285 nm and 545 nm absorbance suggest that V1 may interact with these amino acids. Notably, the second peak indicates insulin polymerization in hexamers (Fig. 1A). In silico analyses showed that Tyr and His interactions with vanadate were not limited (Fig. 1C). In silico studies also revealed that the most probable speciation to vanadate is tetravanadate (cyclotetravanadate), where the interactions involve mainly van der Waals and hydrogen bonds between His5 and Phe1 (Supplementary Table 1 and Fig. 1). Tyr14, Glu21, and Gln4 hydrogen bonds stabilized insulin hexamers with 2 to 6 DV10 with high binding energy (−10.27 to −9.68 kcal/mol; Table 1). DV10 forms a variety of supramolecular assemblies due to its excellent proton-accepting properties, forming hydrogen-bond interactions with proton-donor functional groups [23, 26]. Interestingly, only DV10 presented a characteristic shoulder at about 400 nm, possibly due to its unique electronic transitions or interactions with insulin, which could have implications for insulin stability and activity. At this wavelength, the DV10 dissociation kinetics can be evaluated [30]. The results suggest slow dissociation when insulin is coordinated; however, this point must be corroborated by NMR studies. NMR is also necessary for V1 and its possible oligomerization (V4 and/or V5 species), as well as for DV10 and its possible decomposition into more stable species, which could have distinct biological implications and pharmacological applications [31, 32].

The IR spectrum of V1 and DV10 further supports the presence of specific functional groups that may interact with insulin. For instance, the N—H bending mode appears at 1440 cm⁻¹ for ins-V1 and ins-DV10. The V = O stretching modes are observed at 958 cm⁻¹ for ins-V1 and 970 cm⁻¹ for ins-DV10. Similarly, the V–O–V terminal stretching mode appears at 715 cm⁻¹ for ins-V1 and 742 cm⁻¹ for ins-DV10. The V–O–V in plane bending modes was observed at 550 cm⁻¹ for ins-V1 and around 588 cm⁻¹ for ins-DV10 (Fig. 1B) [33–35].

### Short-Term Effect of Insulin Functionalized with Vanadium on Glycemia and Lipid Serum Profile

The specific aim was to demonstrate the effectiveness of insulin functionalized with V1 or DV10. For that, a TD1-like model was induced in Wistar rats. The TD1 model was induced by alloxan because oxidative stress is generated in Langerhans islets after alloxan administration, leading to beta-cell apoptosis and low insulin levels after the third day [14, 36]. The model was validated by inducing hyperglycemia (blood glucose levels above 400 mg/dL) and hypoinsulinemia (insulin levels below 2 µU/mL). After model validation, treatments began, HUMALOG^®^ 75/25 observed lispro (fast-acting) and lispro-protamine (intermediate-acting) action. Lispro insulin (fast-acting) acts within the first 60 min because it is similar to native human insulin, is safe, and has a receptor-binding affinity indistinguishable from that of native insulin (Fig. 2B and F) [37–39]. Insulin binds to its receptor tyrosine kinase (RTK), eliciting receptor autophosphorylation and activation. RTK phosphorylation begins a series of signaling cascades on the insulin receptor substrate (IRS). The phosphorylation of IRS is sequentially phosphorylated by phosphatidylinositol 3-kinase (PI3K) and, subsequently, Akt/protein kinase B (PKB), which is known as the metabolic arm [3, 40, 41]. In muscle and adipose tissue, the insulin-associated metabolic arm promotes the translocation of glucose transporter-4 (GLUT4) to uptake glucose [42].

Lispro insulin reduced blood glucose levels by 50% within 15 min, reaching a mean of 137 mg/dL at 60 min. Equimolar combinations of ins-V1 showed hypoglycemic effects and pharmacodynamic profiles similar to those of lispro insulin, with no differences at varying hormone molar concentrations. Vanadate had a 19.3% lower hypoglycemic effect. Meanwhile, ins-DV10 also showed hypoglycemic effects, but glycemia was more homogeneous across treated groups, with no significant differences compared to lispro insulin (Fig. 2A and E). The hypoglycemic effect of DV10 was 52.4%, indicating that this molecule possesses biological effects per se, even in the absence of insulin. In addition, plasma insulin levels increased progressively with equimolar combinations of ins-DV10, but the concentrations were lower than those of ins-V1, suggesting slower pharmacokinetics (Fig. 2B and F). Therefore, both ins-V1 and ins-DV10 formulations exhibit insulin-enhancing properties, significantly lowering blood glucose levels compared to untreated diabetic controls (NT-T1D). However, their efficiency varies with formulation, while the Ins-DV10 (5:5) formulation achieved an overall mean glucose of 119.5 mg/dL, significantly lower than the ins-V1 (5:5) mean of 143.8 mg/dL. This suggests that decavanadate provides a more potent synergistic effect with insulin at balanced ratios, likely due to its higher charge density and stronger interaction with the insulin signaling pathway, potentially through more effective inhibition of Protein Tyrosine Phosphatases (PTPases) such as PTP1B [43]. Interestingly, when administered as a standalone agent (without insulin), metavanadate was more effective than decavanadate (133.6 mg/dL vs. 166.1 mg/dL), suggesting that V1 might have better cellular uptake or bioavailability when not complexed with the insulin-protamine.

The lispro-protamine activity was also monitored for 12 h. Protamine is a positively charged protein that binds to insulin hexamers, causing precipitation and resulting in a suspension formulation of insulin [8, 9]. When injected, the protamine/insulin crystals dissolve slowly, delaying the dissociation of insulin hexamers and thus slowing the absorption of insulin monomers into the circulation, as was observed in our results. The lispro-protamine pharmacokinetics showed a maximum peak at 2 h and a slow return at 12 h post-administration. The most striking distinction between the two oxovanadates lies in their temporal action profile. In long-term response models, commercial Lispro insulin exhibits rapid clearance, with glucose levels rebounding to hyperglycemic states (> 280 mg/dL) by the 12-hour mark. In contrast, vanadate-complexed formulations significantly extend the therapeutic window. Notably, equimolar combinations of ins-V1 (5:5 and 3:7) extended the half-life of insulin in the bloodstream for 6 h and its hypoglycemic effect (212.3 mg/dL to 160.0 mg/dL; *p* < 0.01). Meanwhile, almost all ins-DV10 combinations extended insulin’s half-life by eight or more hours. In particular, the ins-DV10 (3:7 and 5:5) formulations showed significantly lower average glucose levels (below 160 mg/dL) than Lispro insulin (*p* = 0.0026 and 0.0095, respectively). This indicates that decavanadate stabilizes the insulin-protamine complex more effectively, or its larger oligomeric structure slows the clearance of the vanadate ions, providing a sustained inhibitory effect on gluconeogenesis and an insulin-sensitizing prolonged impact (Fig. 2D and H).

The pharmacodynamics were also improved, observing a prolonged hypoglycemic effect of ins-V1, and the 5:5 and 3:7 combination maintained lower blood sugar levels for up to 12 h compared to lispro-protamine alone; thereby, higher insulin ratios resulted in better glycemic control, while higher V1 ratios resulted in better lipid control. Ins-DV10 combinations observed a maximal effect at 6 h and maintained glycemia below 200 mg/dL for 12 h while showing lipid control, an essential improvement on lispro-protamine insulin (Fig. 2C and G; Table 2). Studies in STZ-induced diabetic rats treated with either vanadium coordination compounds or inorganic vanadium salts administered for 28 days showed significantly improved hyperglycemia and glucose intolerance, indicating the hypoglycemic capability of vanadium. Bis(2-ethyl-3-hydroxy-4-pyronato)oxovanadium(IV) (BEOV) and bis(3-hydroxy-2-methyl-4-pyronato)oxovanadium(IV) (BMOV) have been used in acute and chronic studies in STZ diabetic rats, demonstrating being more efficient than inorganic vanadium salt [44–46]. Likewise, we informed that decavanadate and decavanadate-metformin possess potent hypoglycemic effects, enhancing insulin activity [14, 15, 17, 18]. Pereira et al. also showed that decavanadate has the highest insulin-like activity compared with pyridine-2,6-dicarboxylatedioxovanadium(V) (V5-dipic), BMOV, amavadine, and oligovanadates from metavanadate administration, in the presence of insulin (10 nM); only decavanadate increases (50%) glucose uptake in rat primary adipocytes [47]. However, lipid control remains controversial. Previously, we also informed that decavanadate and chimeric decavanadates, such as metformin-decavanadate, possess lipidic and glycemic control capabilities [14, 17, 18].

Improving dysglycemia and dyslipidemia depends on insulin function and on V1/DV10 distribution and activity in tissue metabolism, which are influenced by their speciation/decomposition rates. In serum, vanadium levels increased with higher administered doses, but DV10 combined with insulin exhibited higher serum levels than vanadate groups at the same molar combinations (Table 2). It has been informed that the decomposition of vanadium decameric species depends on concentration, pH, and ionic strength [30]. Ramos et al. reported that the half-life time of 10 µM decavanadate in buffers with pH 7–7.5 was between 5 and 10 h [48]. Silva-Nolasco et al. stated that the half-life of sodium decavanadate to 0.5- and 1-mM concentration in DMEM medium at 25 °C was 9 h, and that of metformin-decavanadate was 11 h [49]. Vanadium in plasma is distributed and stored in different tissues in three recognized phases. The first phase is a rapid decline with a half-life of 1 h, followed by a second intermediate decline (t_1/2_ ≈ 26 h), and a third slow decline with a half-life of ≈ 10 days [3]. Therefore, most tissues contain less than 10 ng vanadium/g/g wet weight because vanadium is cleared renally in the first 24 h, reducing to about 30% and, after 12 days, to around 50% [4, 50, 51]. Soares et al. reported that after intravenous administration of metavanadate and decavanadate solutions in *Sparus aurata*, the highest vanadium concentration was observed in blood plasma one hour after exposure, approximately 1000-fold higher than in red blood cells, and after 12 h in the heart [31, 32]. Other studies have shown that after developing STZ-induced hyperglycemia and supplementing with vanadium, it accumulates in the kidney, liver, bone, and pancreas [52, 53]. Previously, we reported that vanadium distribution in control Wistar rats administered orally 5 µM NaVO_3_/kg twice a week for 2 months was as follows: liver < renal cortex < renal medulla < heart < adipose tissue [28]. In the present work, our results showed that vanadium was distributed in the renal cortex, liver, muscle, renal medulla, and heart at 3 and 30 days of treatment; in contrast with other studies, decavanadate and ins-DV10 combinations accumulate less tissue vanadium than metavanadate and ins-V1 combinations (Tables 2 and 3). Additionally, we showed that all treatments administered improved alloxan-induced hepatic and renal damage in the long term, which is also associated with chronic metabolic disorders. Vanadium accumulation did not provide additional hepatic or renal toxic effects (see Supplementary Table 2).

### Short-Term Effect of Insulin Functionalized with Vanadium on Glycogen and Triglyceride Tissue Stores

In tissues, both insulin and vanadium positively influence glycogen synthesis. However, the best results are obtained with ins-DV10 mixes (Fig. 3A–E). Insulin via the metabolic arm modulates glycogen synthase kinase 3β and glycogen synthase activity, and vanadate inhibits protein tyrosine phosphatases 1B and phosphatase and tensin homolog that exert adverse effects on insulin action and glucose metabolism in diabetes models and subjects because it can mimic the 5-coordinate transition state of phosphate formed during the phosphatase catalytic cycle [3, 4, 54]. Vanadate may also cause insulin-like effects by activating a cytosolic kinase that stimulates glucose oxidation [55, 56]. The vanadate oxidation promotes the formation of the pervanadate intermediate, which triggers glucose uptake by increasing the autophosphorylation of the insulin receptor and its substrates, thereby preventing dephosphorylation. The pervanadate species also acts as an insulin enhancer, markedly increasing maximal cell responsiveness by stimulating glucose transport at saturating insulin concentrations [55, 57, 58].

Likewise, insulin regulates lipid homeostasis through the IRS/PI3K/Akt/sterol regulatory element-binding protein 1c (SREBP1c) pathway. Both serum and tissue equilibrium depend on stored and beta-oxidized lipids. Metabolic dysfunction, such as diabetes and hypertriglyceridemia, breaks lipid homeostasis, promotes oversteatosis, and reduces beta-oxidation. Insulin combined with V1 and DV10 restores lipid homeostasis, preventing steatosis (Fig. 3F–J). In concordance with serum concentrations, tissues improve lipid storage at high vanadium concentrations. The putative anti-lipolytic actions of vanadium in murine and human adipocytes, tested with increasing doses of 0.1 to 100 µmol/L, on triglyceride breakdown (lipolysis, release of FFA and glycerol), demonstrated its efficient anti-lipolytic activity [58]. In isolated hepatocytes and adipocytes, sodium metavanadate modulated lipid metabolism by stimulating lipogenesis and suppressing lipolytic activity [59]. Metformin-decavanadate’s lipid-lowering and metabolic regulatory activities have also been observed in insulin-requiring models [14, 17, 18, 60]. Lipid metabolism behavior suggested improvement in tissues, specifically in the energy-obtaining mode, because the rates of hepatic triglyceride synthesis from fatty acid esterification depend on substrate flux and are independent of circulating plasma insulin concentrations [61–64].

### Long-Term Effect of Insulin Functionalized with Vanadium on Glycemia and Lipid Serum Profile

Daily lispro, ins-V1, ins-DV10, V1, and DV10 were administered for one month, gradually improving fasting blood glucose levels, even before dosing. The most effective treatments were V1, DV10, and ins-DV10 (especially the 3:7 and 1:9 combination) that demonstrated a decrease in fasting blood sugar (Fig. 4A and E). The results suggest a pharmacokinetic profile similar to long-acting insulin analogs. This was corroborated by quantifying fasting insulin; ins-V1 and ins-DV10 treatments led to higher fasting insulin concentrations than Lispro treatment (Fig. 4B and F). Remarkably, ins-DV10 combinations 3:7 and 5:5 exhibited insulin levels comparable to those of intact rats, indicating substantial improvement with lispro-protamine insulin and treatment advantages. The experimental model was challenged with an OGTT 15 min after the corresponding dosages. Effectiveness in controlling blood sugar after a glucose load was observed in the ins-V1 and ins-DV10 treatments; even vanadate and decavanadate alone maintained glucose levels similar to those of the lispro treatment. The pharmacodynamics between treatments were similar, but ins-DV10 pharmacokinetics were slower (Fig. 4C-D and G-H). A significant limitation of intermediate insulin treatment is its inability to meet basal insulin needs [65, 66]. The pharmacokinetic profile of our insulin-functionalized formulation, compared with lispro-protamine insulin, demonstrates a stable insulin formulation with a long-lasting pharmacodynamic profile similar to endogenous basal insulin. These properties of the long-acting analogs may also reduce the risk of hypoglycemia, especially nocturnally [6, 8, 9].

### Long-Term Effect of Insulin Functionalized with Vanadium on Glycogen and Triglyceride Tissue Stores

Other advantages of using insulin functionalized with vanadate and decavanadate for prolonged periods include improved lipid profiles and carbohydrate management, particularly by preventing glycosylation (fructosamine levels) and over-flux of FFA and triglycerides (Table 3). Remarkable results are observed in treatment with ins-DV in a 3:7 mix. In addition, all vanadium treatments led to metal accumulation in tissues without exceeding the toxicological threshold, with the same distribution as observed with acute treatment (Table 3). The dosage of vanadium and vanadium compounds has a minimal risk level; e.g., the no-observed-adverse-effect level is 0.12 mg/kg/day for 365 days [4]. None of our treatments exceeded this concentration; thus, vanadium dosage is safe. In tissues at pH ≈ 7 and in an environment with favorable ionic strength, concentration, and oxidative stress, vanadate can form tetravanadate, which stabilizes it and keeps it in cells [67–70]. Meanwhile, decavanadate is thermodynamically unstable at pH values above 6. However, it decomposes slowly, with a half-life of about 9 h at pH ≈ 7.5 in cells, but this depends on compartmentalization. In contrast, under acidic conditions, the compound’s stability changes significantly, as the polyanion can undergo protonation, favoring its speciation and biological activity [19, 21, 48, 67, 71, 72]. Therefore, ins-DV10 combinations showed better glycogen synthesis and low triglyceride storage in all analyzed tissues, highlighting 1:9 and 3:7 mixes. Contrary results are observed in the ins-V1 combinations (Fig. 5). However, further research is needed to fully understand the mechanisms of action and optimize these compounds for clinical application.

In summary, we modified HUMALOG^®^ insulin and functionalized it with V1 or DV10 for the first time. Results suggest that both V1 and DV10 stabilize and promote insulin complexation as hexameric forms. Lispro, the fast-acting insulin, was modified with different molar proportions of ins-V1 or ins-DV10. Also, the pharmacodynamics and pharmacokinetics of lispro-protamine were improved, although the ins-DV10 combinations were more homogeneous than the ins-V1 mixes. Likewise, lipid and carbohydrate homeostasis were ameliorated. In long-term administration, the ins-DV10 combinations showed better glycemic and lipid control and lower tissue glycogen and triglyceride storage than the ins-V1 combinations, particularly the 3:7 and 1:9 ins-DV10 combinations. Remarkably, DV10 treatment alone also improves glucose and lipid management. In conclusion, insulin functionalized with a higher proportion of DV10 demonstrated greater effectiveness in controlling lipid and carbohydrate homeostasis. Thus, it could be considered a treatment strategy for diabetes.

## Supplementary Information

Below is the link to the electronic supplementary material.

Supplementary Material 1 (DOCX 23.5 KB)

Supplementary Material 2 (DOCX 324 KB)

## Data Availability

No datasets were generated or analysed during the current study.
