# Supplementary material for: Enhanced Therapeutic Efficacy of Lispro-Protamine Insulin Via Vanadate and Decavanadate Functionalization in a Type 1 Diabetes Murine Model
Source: Biol Trace Elem Res. 2026 Jan 7;204(6):4299–322. doi: 10.1007/s12011-025-04966-7 (PMC13157437; doi:10.1007/s12011-025-04966-7)
Supplement: Supplementary file 1 — Supplementary Material 1 (DOCX 23.5 KB) [file 12011_2025_4966_MOESM1_ESM.docx]

| **Metabolite** | NT-T1D  (n=5) | Lispro  insulin  (n=5) | Ins-V1  (9:1)  (n=5) | Ins-V1  (7:3)  (n=5) | Ins-V1  (5:5)  (n=5) | Ins-V1  (3:7)  (n=5) | Ins-V1  (1:9)  (n=5) | NH_4_VO_3_  (n=5) | Ins-DV10  (9:1)  (n=5) | Ins- DV10  (7:3)  (n=5) | Ins- DV10  (5:5)  (n=5) | Ins- DV10  (3:7)  (n=5) | Ins- DV10  (1:9)  (n=5) | Decavanadate  (DV10)  (n=5) |
| --- | --- | --- | --- | --- | --- | --- | --- | --- | --- | --- | --- | --- | --- | --- |
| Urea (mg/dL)  R.V. = 40 - 57 | **104±5.5** | **116±3.0** | 41±17.5 | **29.7±1.5** | 42.7±15 | 53±21.5 | **78±14** | 44.3±19.5 | 43±12.5 | **58±19.5** | 48±6 | 56.3±17.5 | **86.3±35** | 57±14 |
| Creatinine (mg/dL)  R.V. = 0.6 – 0.82 | **1.2±0.02** | 0.82±0.2 | 0.79±0.08 | 0.74±0.12 | **0.83±0.1** | 0.78±0.16 | 0.78±0.14 | 0.78±0.1 | 0.74±0.13 | 0.79±0.2 | 0.8±0.06 | 0.77±0.07 | **0.87±0.17** | **0.94±0.1** |
| Sodium (mmol/L)  R.V. = 145 - 150 | **131.7±2.0** | 150±2.0 | 148±1.0 | 149±1.0 | 149±1.0 | 148.6±1.5 | 149±1.5 | 148±1.5 | **151±2.0** | 149.4±2.9 | 147.8±1.5 | 145.8±3.0 | **151±5.2** | 148±1.0 |
| Potassium (mmol/L)  R.V. = 5.5 – 6.0 | **4.8±0.35** | **5.14±0.27** | 5.73±0.05 | 5.7±0.1 | 5.51±0.35 | 5.8±0.1 | 5.71±0.77 | 5.5±0.3 | **5.4±0.43** | 5.5±0.26 | 5.7±0.1 | 5.63±0.1 | 5.81±0.9 | 5.53±0.1 |
| ASAT (UI/L)  R.V. = 150 - 190 | **393±14.5** | **143±4.5** | 154±31 | 152±6.0 | **194±8.0** | **296±99** | 189±45 | **204±31** | 159±18 | 183±36 | **207±15** | **230±100** | 157±33 | **213±48.5** |
| ALAT (UI/L)  R.V. = 60 - 80 | **98.7±6.0** | **96±12** | **105±3.0** | 77±6.0 | **83±3.0** | **177±72** | **86±58** | **114±51** | 79±13 | **101±17** | **90±16** | **90±35** | 75±7 | **86±15** |
| 0ALP (UI/L)  R.V. = 250 - 400 | **747±94.5** | **549±101** | **412±80** | 370±18 | **581±297** | **408±35** | **478±29** | **566±206** | 373±109 | **684±64** | **585±306** | 322±191 | **457±179** | **441±197** |
| Total Bilirubin (mg/dL)  R.V. = 0.45 – 0.65 | **1.02±0.16** | 0.48±0.13 | **0.38±0.2** | **0.22±0.07** | **0.38±0.1** | **0.66±0.2** | **0.43±0.04** | **0.21±0.1** | 0.5±0.14 | **0.7±0.12** | 0.58±0.2 | **0.40±0.09** | **0.41±0.1** | 0.45±0.09 |

**Table S2.** Serum biomarkers of hepatic and renal damage.

The results shown are the average of 5 separate experimental animals ± SEM. R.V. Reference value from intact control, male Wistar rat of the same age as the experimental groups. Reference values ​​outside the biological range are shown in bold.
