# Supplementary material for: Enhanced Therapeutic Efficacy of Lispro-Protamine Insulin Via Vanadate and Decavanadate Functionalization in a Type 1 Diabetes Murine Model
Source: Biol Trace Elem Res. 2026 Jan 7;204(6):4299–322. doi: 10.1007/s12011-025-04966-7 (PMC13157437; doi:10.1007/s12011-025-04966-7)
Supplement: Supplementary file 2 — Supplementary Material 2 (DOCX 324 KB) [file 12011_2025_4966_MOESM2_ESM.docx]

**Docking Results**

***Table S1****. Docking results. Binding energies for the best molecular poses of cyclotetravanadate in complex with Lys-Pro Insulin*

| Compound | Chain | Binding Energies (Kcal/mol) | Interactions |
| --- | --- | --- | --- |
| Cyclotetravanadate + Insulin Lys-Pro | L, H | -8.5 | Hydrogen bond (6), Phe1, **Gln4, His5**, ILE10, Tyr16, Gly20, Glu21**, Tyr26**, Lys28 |
|  | H, G, B | -7.64 | Hydrogen bond (2), **His5**, Leu6, Cys6, Cys7, Leu11, His10, Ile10, **Cys11**, Ala14, Leu16, Leu17, |
|  | F, C | -7.08 | Hydrogen bond (5**), Phe1**, Val2, **Asn3**, Gln4 |
|  | J, K | -6.99 | Hydrogen bond (2), His5, Ile10, **Leu13**, Leu17, Val18, Gly20, **Glu21** |
|  | I, L, H | -6.70 | Hydrogen bond (2), **His5**, *Leu6, Cys6, His10,* ***Cys11****, Leu11, Ala14, Leu16, Leu17* |
|  | E, F | -6.69 | Hydrogen bond (3), **Ile2**, **Val3**, Tyr19, **Thr27**, Lys28, Pro29 |
|  | H, I, J | -6.61 | Hydrogen bond (2) Phe1, Val2, Val3, Asn3, **Asn3**, Gln4, **Cys7**, Thr8, Cys7 |
|  | G, H, L | -6.5 | Hydrogen bond (5) Ile2, Val3, **Gln4**, Gly8, Tyr16, **Glu21**, Thr26, **Thr27** |
|  | A, B | -6.41 | Hydrogen bond (3) Gly1, **Ile2**, **Val3**, Tyr19, **Thr27**, Lys28, Tyr29, Pro29 |
|  | F, B | -6.41 | Hydrogen bond (1**) Phe1**, Gln4, His5, Ile10, Gly20, Lys28, Pro29 |
|  | G, H, F | -5.92 | Hydrogen bond (5) **Phe1,** Gln4, **His5, Ile10, Lys28** |

The docking analysis was carried out using 200 Genetic Algorithm poses to explore all possible interaction sites between the cyclotetravanadate and the protein. In Table 1, the top 11 poses of cyclotetravanadate interacting with different chains are reported. The binding energies ranged from -8.5 to -5.92 kcal/mol. The interactions involve mainly van der Waals and hydrogen bonds between His5 and Phe1 from different protein chains. In Figure S1, the 11 poses of cyclotetravanadate interacting with varying insulin chains are shown.


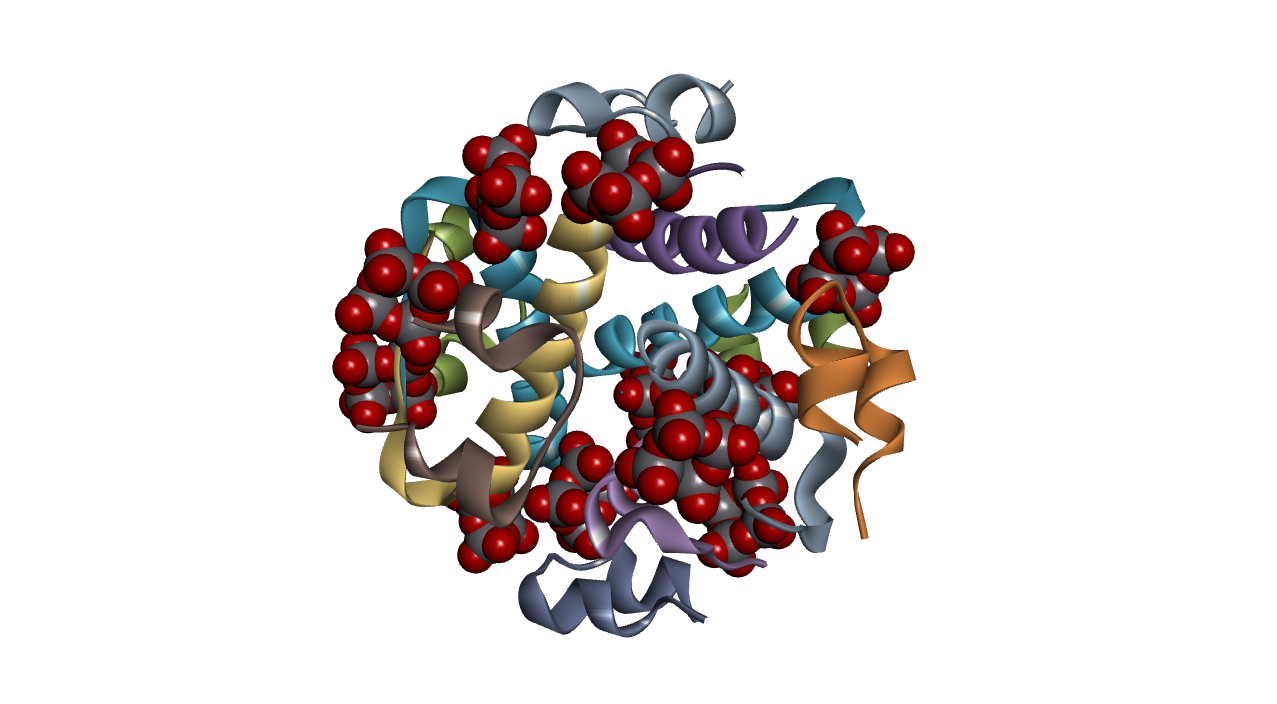


**Figure S2**. Interactions of cyclotetravanadate with insulin. *Chain A: Purpure, Chain B: pink, Chain C: Green, Chain D: orange, Chain E: blue, Chain F: gray, Chain G: dark blue, Chain H: dark green, Chain I: cyan, Chin J: gold, Chain K: pale blue, Chain L: pale red*
